# A Comparative Study of Four Kinds of Adaptive Decomposition Algorithms and Their Applications

**DOI:** 10.3390/s18072120

**Published:** 2018-07-02

**Authors:** Tao Liu, Zhijun Luo, Jiahong Huang, Shaoze Yan

**Affiliations:** 1 State Key Laboratory of Tribology, Department of Mechanical Engineering, Tsinghua University, Beijing 100084, China; liutao13@mails.tsinghua.edu.cn (T.L.); luozj16@mails.tsinghua.edu.cn (Z.L.); jiahong-14@mails.tsinghua.edu.cn (J.H.); 2 High-Tech Institute, Qingzhou 262500, China

**Keywords:** signal processing, non-stationary signal, narrow-band signal, adaptive decomposition algorithm

## Abstract

The adaptive decomposition algorithm is a powerful tool for signal analysis, because it can decompose signals into several narrow-band components, which is advantageous to quantitatively evaluate signal characteristics. In this paper, we present a comparative study of four kinds of adaptive decomposition algorithms, including some algorithms deriving from empirical mode decomposition (EMD), empirical wavelet transform (EWT), variational mode decomposition (VMD) and Vold–Kalman filter order tracking (VKF_OT). Their principles, advantages and disadvantages, and improvements and applications to signal analyses in dynamic analysis of mechanical system and machinery fault diagnosis are showed. Examples are provided to illustrate important influence performance factors and improvements of these algorithms. Finally, we summarize applicable scopes, inapplicable scopes and some further works of these methods in respect of precise filters and rough filters. It is hoped that the paper can provide a valuable reference for application and improvement of these methods in signal processing.

## 1. Introduction

At present, a great number of scholars conduct investigations about adaptive decomposition algorithms. It is difficult to find a rigorous definition of the adaptive decomposition algorithm; however, we think that such a type of method can form a series of sparse representations in the decomposition process, which is different with “rigid” methods, such as the Fourier or wavelets transforms, corresponding to the use of some basis (or frame) designed independently of the processed signal [1,2]. As many kinds of signals in engineering problems are non-linear and non-stationary, such as fault signals of mechanical equipment [3,4,5,6,7,8], some modal test signals [9], acoustic signals of non-destructive testing [10,11] and condition monitoring signals for rail track [12,13,14], the adaptive decomposition algorithm has superiority for analyzing these signals, because of decomposition flexibility.

Currently, empirical mode decomposition (EMD), empirical wavelet transform (EWT), variational mode decomposition (VMD) and Vold–Kalman filter order tracking (VKF_OT) are popular adaptive decomposition algorithms. These methods show excellent capacity of processing non-linear and non-stationary signals. Some important improvements have been done for EMD in some other algorithms such as complementary ensemble empirical mode decomposition (CEEMD), complementary ensemble empirical mode decomposition with adaptive noises (CEEMDAN) and improved complementary ensemble empirical mode decomposition with adaptive noises (improved CEEMDAN), which are more competent at processing non-linear and non-stationary signals. However, these adaptive decomposition algorithms have their own characteristics, which affect performances. Therefore, a comparative study that illustrates factors to consider when applying these adaptive decomposition algorithms will be welcome to researchers processing non-linear and non-stationary signals. Techniques that further process decomposition results of these methods are also valuable, so we will summarize this kind of technology in this paper. Furthermore, we present some further works that can be done for these methods in this paper, hoping some improved versions can be proposed to solve problems when processing non-linear and non-stationary signals.

The remainder of this paper is organized as follows. Section 2 presents the principles and influence factors of the decomposition result, improvements of algorithms deriving from EMD and investigations of theory and application of EMD. Section 3, Section 4 and Section 5 present principles, advantages and disadvantages of EWT, VMD and VFK_OT and investigations of theory and application of these algorithms. Section 6 summarizes the characteristics of these adaptive decomposition methods and points out areas for future work.

## 2. Algorithms Deriving from Empirical Mode Decomposition

In 1998, Huang [1] proposed EMD, which takes intrinsic mode functions (IMFs) that are narrow-band components to act as basic functions, to obtain sparse representation of analyzed signals, as mentioned above. Decomposing signals into narrow-band components can result in advantages of time-frequency analysis. For example, multi-component signals can be decomposed into amplitude and frequency modulated (AM and FM) components, which makes it feasible for obtaining instantaneous frequency (IF) and instantaneous amplitude (IA) by using Hilbert transform (HT). Valuable components can be extracted by EMD, which is helpful for obtaining the necessary features of signals. Therefore, numerous researches of theory and application were done for EMD. Among these works, ensemble empirical mode decomposition (EEMD), complementary ensemble empirical mode decomposition (CEEMD), and complementary ensemble empirical mode decomposition with adaptive noise (CEEMDAN) are remarkable. Therefore we present the principles of these methods, and the corresponding superiorities over EMD. The issue of the limitation of frequency resolution of these algorithms and the influence of sampling frequency for decomposition results are discussed to provide a reference for employing these algorithms.

### 2.1. Empirical Mode Decomposition

#### 2.1.1. Principle of Empirical Mode Decomposition

EMD [1] decomposes a signal f(t) into a small number of IMFs. To be considered as an IMF, a signal must fulfill two conditions: (1) the number of extrema (maxima and minima) and the number of zero-crossings must be equal or differ at most by one; and (2) the local mean, defined as the mean of the upper and lower envelopes (The definition of “envelope” can be found in Ref. [1]), must be zero. The algorithm can be described as follows [1]:

(1) Set *k* = 0, and find all extrema of $r_{0}(t)=f(t)$.
(2) Interpolate between minima (maxima) of $r_{k}(t)$ to obtain the lower (upper) envelope $e_{\min}(t)$ ($e_{\max}(t)$).
(3) The mean envelope is calculated by,
  (1) $$m(t)=(e_{\min}(t)+e_{\max}(t))/2$$
(4) The IMF candidate is obtained by,
  (2) $$d_{k+1}(t)=r_{k}(t)-m(t)$$
(5) Repeat Steps (2)–(4) on $d_{k+1}(t)$, until m(t) is close to zero. Then $d_{k+1}(t)$ is an IMF noted as $c_{k+1}(t)$.
(6) Compute the residue by
  (3) $$r_{k+1}(t)=f(t)-c_{k+1}(t)$$
  and do k=k+1.
(7) Residue $r_{k+1}(t)$ is taken as f(t), and repeat Steps (1)–(6) to generate the next IMF and residue, until the final r(t) satisfies the predefined stopping criterion.
  Therefore, the original signal f(t) can be represented by the following formula:
  (4) $$f(t)=\sum_{i=1}^{n}c_{i}(t)+R(t)$$
  where $c_{i}(t)$ is the *i*th IMF and R(t) is the final residue.

The distribution of extreme values of a signal depends on the IA and IF of corresponding mono-components. It can learn from the principle of EMD that EMD utilizes it to extract IMFs in the process of sifting and iteration. Therefore, the method inevitably suffers from limitations in some domains, such as frequency resolution and influence of sampling frequency. These issues are presented in the following sections.

#### 2.1.2. Limitation of Frequency Resolution

Frequency resolution is important for the adaptive decomposition algorithm, as it is a crucial parameter determining the scope of application. Refs. [15,16,17,18] revealed that frequency resolution was related to the number of sifting iterations, stopping criterion threshold setting and the amplitude ratio between different mono-components. For stopping criterion threshold setting, at present, there is no authoritative statement, which may result from EMD’s lack of theoretical basis, and the parameter is set according to experience of a specific question [16]. Refs. [17,18] tried to improve frequency resolution of EMD using different masking operations. For the amplitude ratio between different mono-components, Ref. [15] pointed out the frequency resolution would decrease when amplitude ratio is greater than a threshold. 100 was taken as a reasonable number of sifting iterations in [15]. A great amount of computation work will be done with several iterations greater than 100, and we set this parameter as 2000, which may not be an ideal choice in a specific question. In the following section, research is performed concerning frequency resolution of EMD with open source codes, setting the number of sifting iterations as 2000, setting the stopping criterion threshold as 0.05, and this parameter is also set as 2000 in the following algorithms deriving from EMD.

As indicated in Ref. [15], for a reasonable number of iterations, when the ratio between a relatively low frequency and a relatively high frequency is larger than 0.75, the two components of a signal cannot be separated. To illustrate the conclusion, we construct a sample signal *f_sig_*_1_,

(5) $$\begin{array}{c} f_{sig1}=s_{1}(t)+s_{2}(t)+s_{3}(t)\\ s_{1}(t)=\sin(75\times 2\pi t),0\leq t\leq 1\\ s_{2}(t)=\sin(100\times 2\pi t),0\leq t\leq 1\\ s_{3}(t)=\sin(200\times 2\pi t),0\leq t\leq 1 \end{array}$$

**Figure 1:**
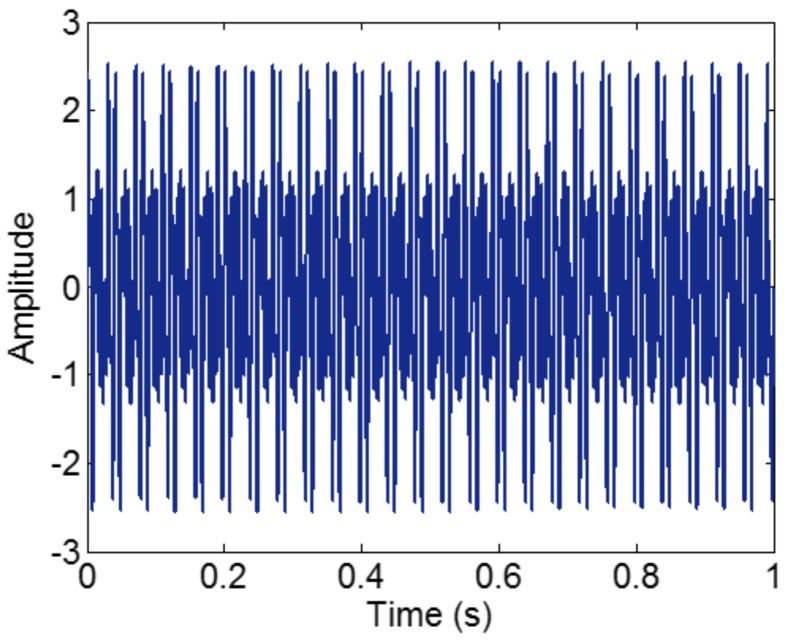
The waveform of the sample signal *f_sig_*_1_ between in time domain.

**Figure 2:**
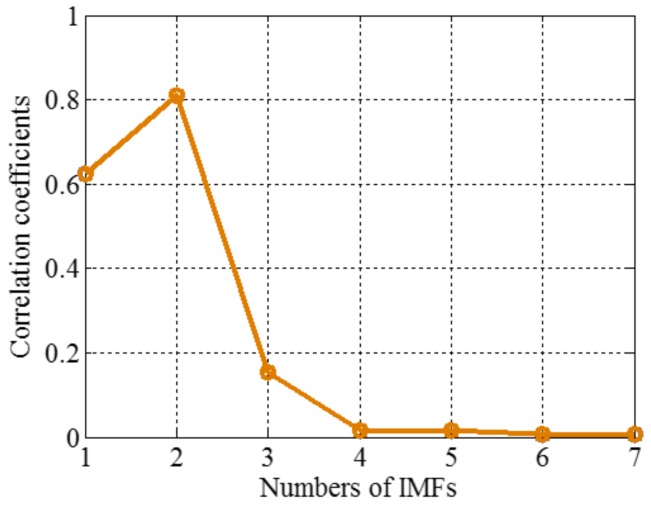
The coefficients of correlation different IMFs and the sample signal.

**Figure 3:**
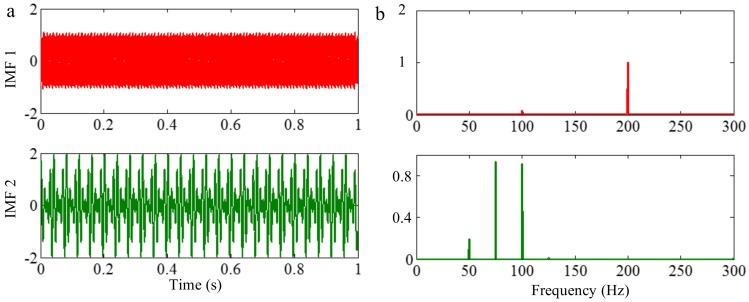
The waveforms of the IMFs 1 and 2 of *f_sig_*_1_ and the corresponding Fourier spectrums: (**a**) waveforms and (**b**) Fourier spectrums.

The sampling frequency is 2 kHz. When the amplitudes of tone components are equal, different components tend to be isolated, so we set it as 1 in components of the sample signal *f_sig_*_1_. The waveform of the sample signal *f_sig_*_1_ in the time domain is presented in Figure 1. Ref. [19] reveals that greater correlation coefficients lead to more important corresponding IMFs for the original signals. Therefore, we pick out the IMFs, whose correlation coefficients with the sample signal *f_sig_*_1_ are greater than 0.2. The coefficients of correlation between different IMFs and the sample signal *f_sig_*_1_ are shown in Figure 2. The correlation coefficients of IMFs 1 and 2 are greater than 0.2, so these IMFs are kept, and shown in Figure 3. As illustrated in Figure 3, the component *s*_3_ of *f_sig_*_1_ is extracted. IMF2 includes the components *s*_1_ and *s*_2_, which mix together, as shown in Figure 3b. The decomposition result above demonstrates the conclusion about frequency resolution of EMD. Furthermore, this conclusion just tells us that, when the ratio is greater than 0.75, the two tones will be taken as a single component, for a reasonable number of iterations.

#### 2.1.3. Influence of Sampling Frequency on Decomposition Result

**Figure 4:**
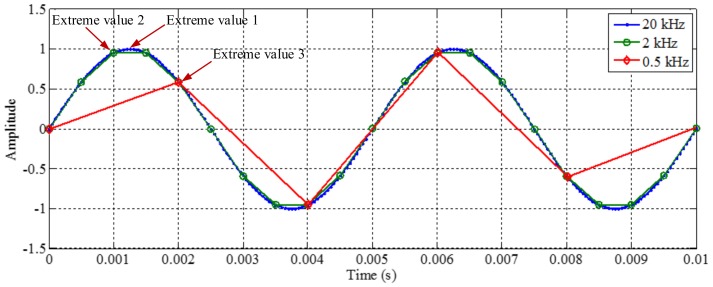
The distributions of extreme values of a signal of 200 Hz with sampling frequencies of 0.5, 2 and 20 kHz.

**Figure 5:**
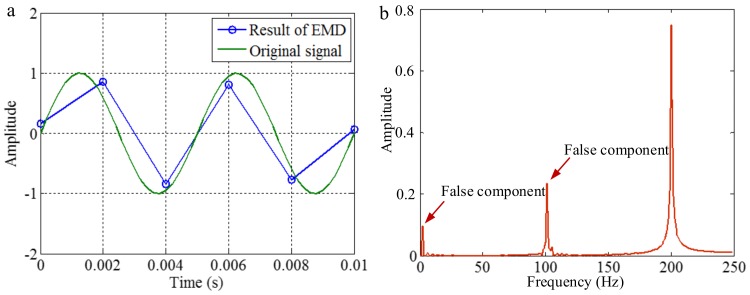
The EMD result of the signal of 200 Hz with a sampling frequency of 0.5 kHz and the corresponding Fourier spectrum: (**a**) the results of EMD and (**b**) the Fourier spectrum.

**Figure 6:**
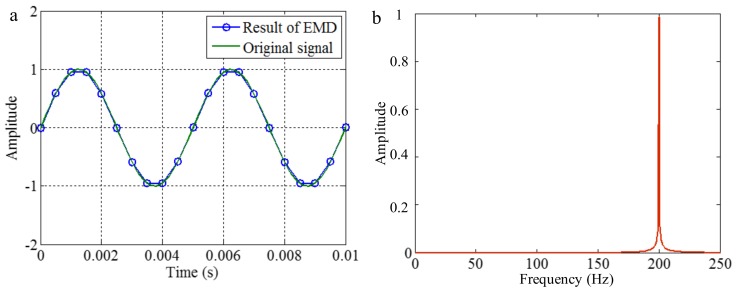
The EMD result of the signal of 200 Hz with a sampling frequency of 2 kHz and the corresponding Fourier spectrum: (**a**) the result of EMD and (**b**) the Fourier spectrum.

As mentioned above, EMD utilizes the distribution of extreme values of a signal to extract IMFs in a process of sifting and iteration. Generally, the distribution of extreme values depends on the IF and the IA of corresponding mono-components of the signal. However, for discrete signals, the true extreme value may be different with the theoretical value. Increasing the sampling frequency is advantageous for decreasing the difference, as shown in Figure 4. The first maximum extreme value of the signal of 200 Hz is in a time of 0.00125 s, and the value is 1. With different sampling frequencies, the times of the extreme value are 0.00125, 0.001 and 0.002 s, and the corresponding values are about 1, 0.95 and 0.58, corresponding to sampling frequencies of 0.5, 2 and 20 kHz. Some false components may be generated from the error of the envelope calculation of cubic spline interpolation and calculation of mean value for the extreme values. To demonstrate it, we apply EMD on the signals with sampling frequencies of 0.5 and 2 kHz, and the signal with a sampling frequency of 20 kHz is taken as the original continuous signal. The decomposition results are shown in Figure 5 and Figure 6, respectively. As shown in Figure 5, when the sampling frequency is 0.5 kHz, the result of EMD is different to the original signal, and false components occur, as can be seen in Figure 5b. It is deduced that, because the sampling frequency is not great enough, based on the extreme values, the envelope calculation of cubic spline interpolation and the further calculation of the mean value cannot generate the original signal. As shown in Figure 6, when the sampling frequency is 2 kHz, the result of EMD corresponds to the original signal. It is deduced that, because the sampling frequency is great enough, based on the extreme values, the envelope calculation of cubic spline interpolation and the calculation of the mean value can generate the original signal in sifting and iteration process.

The conclusion above suggests that, when EMD is applied to the process signal, a relatively higher sampling frequency is advantageous for generating correct decomposition result. Otherwise, an insufficient sampling frequency will result in false components occurring in IMFs.

#### 2.1.4. Phenomenon of Mode-Mixing Caused by Intermittent Signals

In the process of an IMF generation, EMD extracts the component with the highest frequency in every time section. Therefore, every component cannot be intermittence; otherwise, mode-mixing will occur. To demonstrate this, we construct a sample signal *f_sig_*_2_,

(6) $$\begin{array}{c} f_{sig2}=s_{1}(t)+s_{2}(t)+s_{3}(t)\\ s_{1}(t)=\left\{ \begin{array}{l} 0\\ \sin(50\times 2\pi t)\\ 0 \end{array}\right.\begin{array}{l} 0\leq t\leq 0.1\\ 0.1<t\leq 0.6\\ 0.6<t\leq 1 \end{array},\\ s_{2}(t)=\left\{ \begin{array}{l} 0\\ \sin(100\times 2\pi t)\\ 0 \end{array}\right.\begin{array}{l} 0\leq t\leq 0.2\\ 0.2<t\leq 0.7\\ 0.7<t\leq 1 \end{array},\\ s_{3}(t)=\left\{ \begin{array}{l} 0\\ \sin(200\times 2\pi t)\\ 0 \end{array}\right.\begin{array}{l} 0\leq t\leq 0.3\\ 0.3<t\leq 0.8\\ 0.8<t\leq 1 \end{array} \end{array}$$

**Figure 7:**
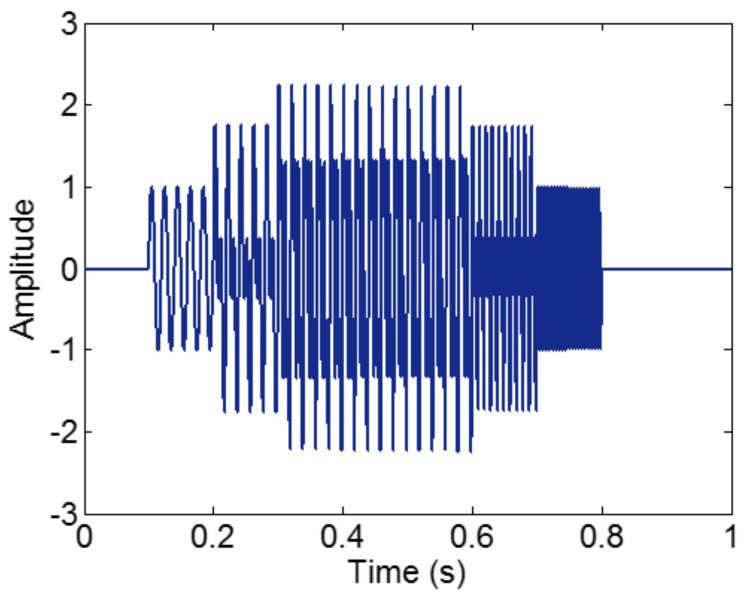
The waveform of the sample signal *f_sig_*_2_ in time domain.

**Figure 8:**
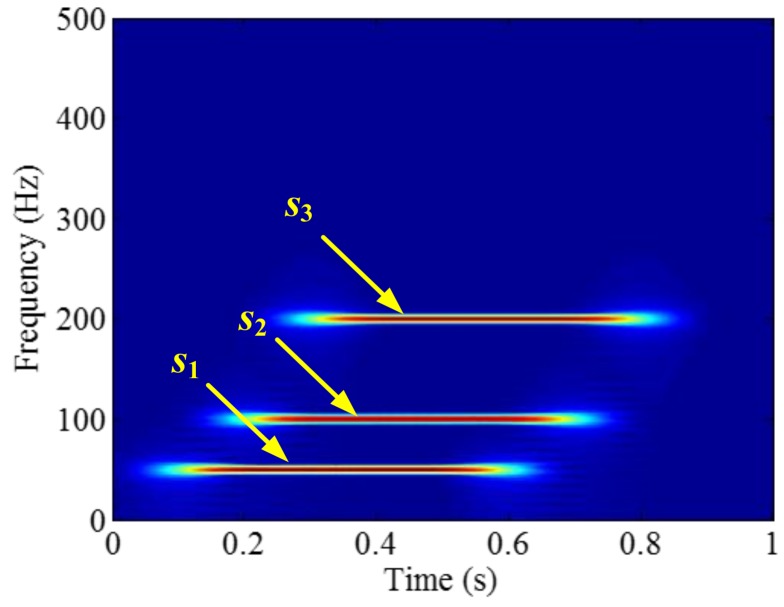
The STFT of the sample signal *f_sig_*_2_.

**Figure 9:**
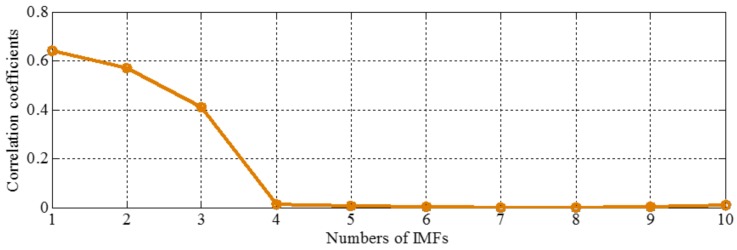
The coefficients of correlation between different IMFs and the sample signal *f_sig_*_2_.

**Figure 10:**
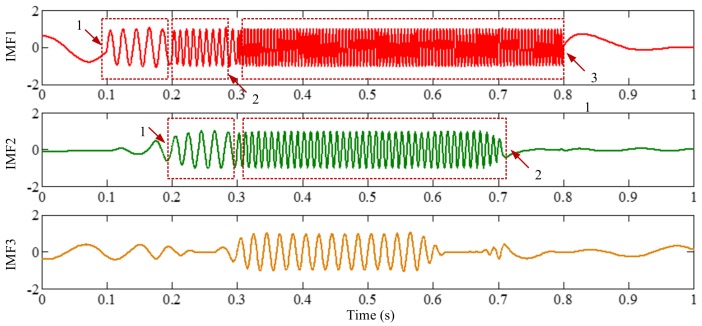
The waveforms of the IMFs 1–3.

**Figure 11:**
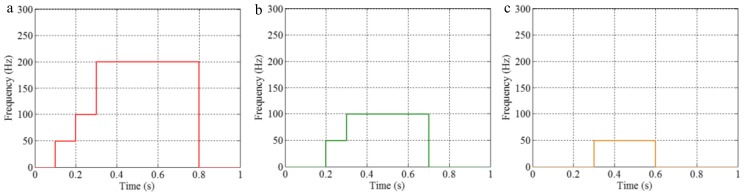
The ideal time-frequency distributions of the IMFs 1–3: (**a**) IMF 1; (**b**) IMF 2 and (**c**) IMF 3.

**Figure 12:**
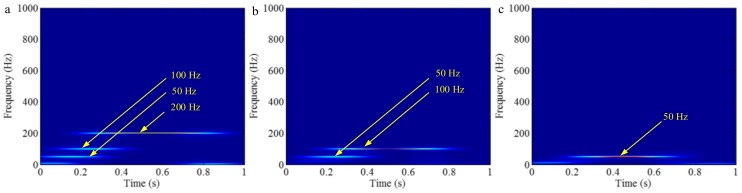
The STFT representations of the IMFs 1–3: (**a**) IMF 1; (**b**) IMF 2 and (**c**) IMF 3.

The sampling frequency is 2 kHz. The waveform of the sample signal *f_sig_*_2_ in the time domain is presented in Figure 7, and the corresponding short-time Fourier transform (STFT) representation is shown in Figure 8. We pick out the IMFs, whose correlation coefficients with the sample signal *f_sig_*_2_ are greater than 0.2. The coefficients of correlation between different IMFs and the sample signal *f_sig_*_2_ are shown in Figure 9. The correlation coefficients of IMFs 1, 2 and 3 are greater than 0.2, so these IMFs are kept, and shown in Figure 10. If the conclusion mentioned above is correct (in the process of an IMF generation, EMD extracts the component with the highest frequency in every time section), the time-frequency distributions of IMFs 1–3 should be as follows: for IMF 1, the frequency of signal in time interval [0.1 0.2] s is 50 Hz; and the frequency of signal in time interval [0.2 0.3] s is 100 Hz; and the frequency of signal in time interval [0.3 0.8] s is 200 Hz. For IMF 2, the frequency of signal in time interval [0.2 0.3] s is 50 Hz; the frequency of signal in time interval [0.3 0.7] s is 100 Hz; for IMF 3, the frequency of signal in time interval [0.3 0.6] s is 50 Hz, as shown in Figure 11. As can be seen in Figure 10, the frequencies in different time sections of IMFs 1 and 2 signed by different red rectangles seem different. To make the time-frequency distribution visible, we do STFT for the IMFs 1–3, as shown in Figure 12, which verifies the ideal time-frequency distributions of IMFs 1–3. Therefore, our deduction is correct.

As revealed in the discussion above, if components of a signal are intermittent, mode-mixing will occur. To resolve this problem, EEMD was proposed.

### 2.2. Ensemble Empirical Mode Decomposition

#### 2.2.1. Resolving the Problem of Mode-Mixing Caused by Intermittent Signals

**Figure 13:**
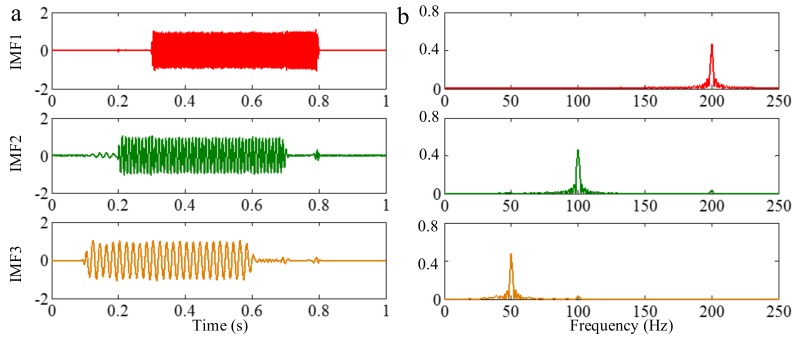
The waveforms of the IMFs 1–3 and the corresponding Fourier spectrums: (**a**) waveforms and (**b**) Fourier spectrums.

Wu and Huang [19] proposed EEMD, which is a marked milestone in the development of EMD. White noise can provide a uniformly distributed scale in the time-frequency space. It can provide similar scales of reference gridings to automatically associate with the intrinsic oscillations in the signal with different scales. Therefore, all the intrinsic oscillations become continuous in the whole signal. As mentioned above, EMD extracts the component with the highest frequency in every time section. Since no intermittence occurs in each intrinsic oscillation, the mode-mixing caused by intermittent signals can be avoided. Afterwards, the mean operation “forces” the mode to stick to the original signal in those portions where new extrema are created, while it remains unmodified in the rest of the signal (where no creation of extrema occurred). Therefore, the mode-mixing caused by intermittent signals is solved. To illustrate this, we employ EEMD on the sample signal *f_sig_*_2_. According to the coefficients of correlation between different IMFs and the sample signal *f_sig_*_2_, we extract valuable IMFs 1–3, and present them in Figure 13. As can be seen in Figure 13, the mode mixing caused by intermittent signals is resolved (especially in Figure 13b).

#### 2.2.2. Principle of Ensemble Empirical Mode Decomposition

In EEMD [19], the “true” modes are defined as the average of the corresponding IMFs obtained from an ensemble of the original signal plus white noise with different strengths. Let *f* be the analyzed signal. The principle of EEMD can be described as follows:

(1) Signals $f_{i}(t)$ are generated by
  (7) $$f_{i}(t)=f(t)+\beta \omega^{\left(i\right)}(t)$$
  where β is the variance of added white noise, and $\omega^{\left(i\right)}(t)$ (*i* = 1, ..., and *i* is the number that EMD is conducted) denotes a zero mean unit variance white noises N(0,1).
(2) Employ EMD to decompose completely each $f_{i}(t)$, and obtain the IMFs $d_{k}^{i}(t)$ (*k* = 1, ..., *k* is the number of IMFs of EMD).
(3) Calculate each final IMF by,
  (8) $$c_{k}(t)=\frac{1}{I}\sum_{i=1}^{I}d_{k}^{i}(t)$$
  where $c_{k}(t)$ (*k* = 1, ..., *k* is the number of IMFs of EEMD) is the *k*th IMF of EEMD.

The extraction of every $d_{k}^{i}(t)$ requires a different number of sifting iterations in EMD.

#### 2.2.3. Limitation of Frequency Resolution

**Figure 14:**
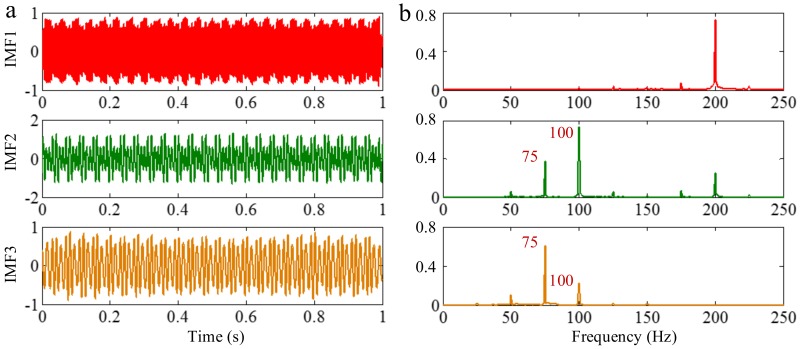
The waveforms of the IMFs 1–3 of *f_sig_*_1_ and the corresponding Fourier spectrums: (**a**) waveforms and (**b**) Fourier spectrums.

Because EEMD derives from EMD, they suffer a similar frequency resolution. When the ratio between a relatively low frequency and a relatively high frequency is larger than 0.75, the two components of a signal cannot be separated by EEMD, for a reasonable number of iterations. To demonstrate this, we decompose the sample signal *f_sig_*_1_ by EEMD, and the valuable IMFs are shown in Figure 14. As illustrated in Figure 14b, the components of 75 and 100 Hz cannot be separated. Therefore, this basic limitation of EMD is also suitable for EEMD.

#### 2.2.4. Influence of Sampling Frequency on Decomposition Result

**Figure 15:**
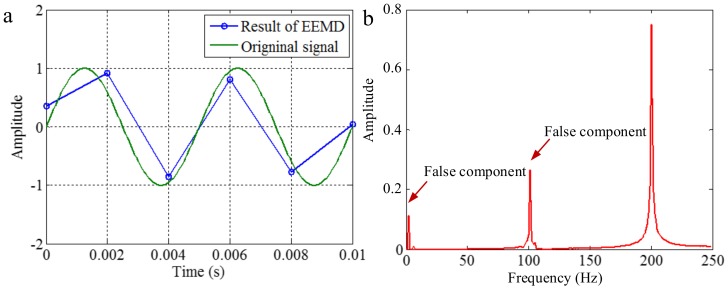
The EEMD result of the signal of 200 Hz with a sampling frequency of 2 kHz and the corresponding Fourier spectrum: (**a**) the result of EEMD and (**b**) the Fourier spectrum.

As mentioned above, the decomposition result of EMD is influenced by the sampling frequency. The similar conclusion can also be obtained for EEMD. To illustrate this, we employ EEMD on a signal of 200 Hz with a sampling frequency of 0.5 kHz. IMF 1 is the valuable IMF for the decomposition result and is shown in Figure 15. As can be seen in Figure 15, false components also occur as a result of EEMD. Therefore, when the sampling frequency is not great enough for EEMD, correct extreme values cannot be obtained, which results in the envelope calculation of cubic spline interpolation and the calculation of mean value for the extreme values not being able to generate the original signal, either. A higher sampling frequency is also welcome in the process of EEMD.

### 2.3. Complementary Ensemble Empirical Mode Decomposition

Although EEMD can avoid mode-mixing resulting from intermittent signals, which is crucial in the application of adaptive decomposition algorithms, adding white noise can introduce residue into the signal reconstructed by decomposition results of EEMD. The residue of the added white noise in EEMD can be extracted from the mixture of data and white noise via the ensemble IMFs with the positive added white noise (it should be noted that the residue mentioned this section is defined as the difference between the original and the reconstructed signals, and this is different from the residue (or trend) generated in the iterative calculation process of EMD). To suppress the residue, Yeh and Shieh [20] proposed CEEMD.

In CEEMD, white noise is added in pairs to the analyzed signal (i.e., one positive and one negative) to generate two sets of ensemble IMFs. Therefore, two mixtures composed of the original data and added noise can be derived by

(9) $$\left[\begin{array}{l} M_{1}\\ M_{2} \end{array}\right]=\left[\begin{array}{cc} 1 & 1\\ 1 & -1 \end{array}\right]\;\left[\begin{array}{l} S\\ N \end{array}\right]$$

where *S* is the original data; *N* is the added white noise; *M*_1_ is the sum of the original data with positive noise, and *M*_2_ is the sum of the original data with the negative noise.

**Figure 16:**
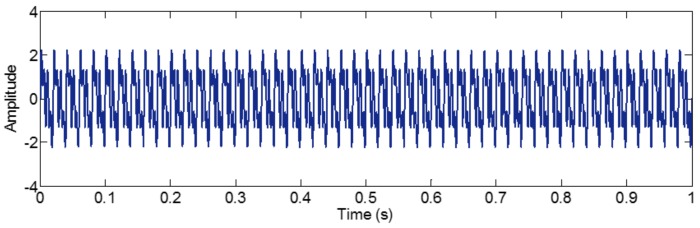
The waveform of the sample signal *f_sig_*_3_.

Then, the ensemble IMFs obtained from those positive mixtures contribute to a set of IMFs with positive residues of the added white noises. Similarly, the ensemble IMFs obtained from those negative mixtures contribute to another set of ensemble IMFs with negative residue of the added white noises. Thus, the final IMF is the ensemble of both the IMFs with the positive and negative noises. This operation can suppress the residue result from adding the white noise. To illustrate this, we construct a sample signal *f_sig_*_3_, and it is shown in Figure 16,

(10) $$\begin{array}{c} f_{sig1}=s_{3}(t)+s_{2}(t)+s_{3}(t)\\ s_{1}(t)=\sin(50\times 2\pi t),0\leq t\leq 1\\ s_{2}(t)=\sin(100\times 2\pi t),0\leq t\leq 1\\ s_{3}(t)=\sin(200\times 2\pi t),0\leq t\leq 1 \end{array}$$

**Figure 17:**
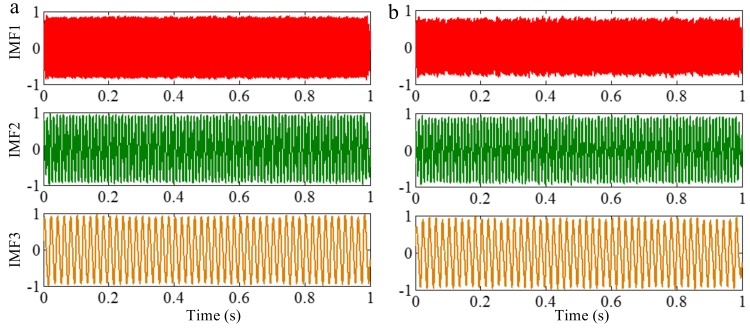
The waveforms of the IMFs 1–3 of *f_sig_*_3_ by EEMD and CEEMD: (**a**) EEMD and (**b**) CEEMD.

**Figure 18:**
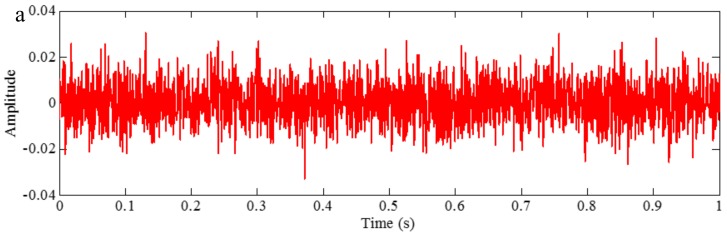

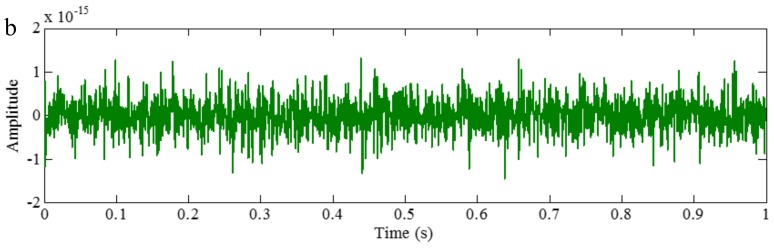
Residues of added white noises derived by EEMD and CEEMD: (**a**) EEMD and (**b**) CEEMD.

We employ EEMD and CEEMD to process the signal *f_sig_*_3_. The coefficients of correlation between different IMFs and the sample signal *f_sig_*_3_ show that IMFs 1–3 are valuable IMFs in EEMD and CEEMD. We present these IMFs in Figure 17. A visual comparison of the results from EEMD and CEEMD shows almost no significant difference. However, differences between the reconstructed signals via the IMFs obtained by EEMD and CEEMD and the original signal are very large. There is a significant different between the final residues derived from EEMD and CEEMD, defined as the differences between the original and the reconstructed signals and are shown in Figure 18. While the residue from EEMD has an average amplitude of around 0.03, the corresponding residue from CEEMD has an average amplitude close to 0 (of the order of 10^−15^). Such an error could be very well attributed to the numerical error generated in the calculation. Thus, CEEMD can improve the decomposition results by removing the residue of the added white noise.

### 2.4. Complementary Ensemble Empirical Mode Decomposition with Adaptive Noise

The computation quantity of an algorithm is an important performance index. As shown in Figure 2 and Figure 9, some useless IMFs are generated in EMD and EEMD, which degrades performance of these algorithms. Therefore, reducing the number of these useless IMFs is advantageous for improving the computation efficiency of these techniques. Torres [21] proposed CEEMDAN, and Colominas [22] proposed an improved version of CEEMDAN. Fewer IMFs may be generated on the premise of successfully separating different components of a signal by using the two algorithms, which can reduce the computational cost.

#### 2.4.1. Principle of Complementary Ensemble Empirical Mode Decomposition with Adaptive Noise

In CEEMDAN, the decomposition modes will be noted as $\tilde{d_{1}}$ and is proposed to calculate a unique first residue as:

(11) $$r_{1}(t)=f(t)-\tilde{d_{1}}(t)$$

where $\tilde{d_{1}}$ is obtained in the same way of EEMD. Then, the first EMD mode is computed over an ensemble of *r*_1_ plus different realizations of a given noise obtaining $\tilde{d_{1}}$ by an averaging calculation. The next residue is defined as: $r_{2}(t)=r_{1}(t)-\tilde{d_{2}}(t)$. This procedure continues with the rest of the modes until reaching the stopping criterion.

The operator $E_{j}(\cdot )$ is defined which generates the *j*th mode obtained by EMD. $\omega^{i}$ is denoted as the white noise with N(0,1). If *f*(*t*) is the analyzed signal, the method can be described by the following steps [21]:

(1) *I* realizations $f(t)+\varepsilon_{0}\omega^{i}(t)$ are decomposed by EMD to obtain their first modes by
  (12) $$\tilde{d_{1}}(t)=\frac{1}{I}\sum_{i=1}^{I}d_{1}^{i}(t)=\bar{d_{1}}(t)$$
(2) At the first stage (*k* = 1), the first residue is calculated as in Equation (11):
  (13) $$r_{1}(t)=f(t)-\tilde{d_{1}}(t)$$
(3) Decomposition of realizations $r_{1}(t)+\varepsilon_{1}E_{1}(\omega^{i}(t)),i=1,\ldots ,I$ is done, until their first EMD mode. The second mode is defined as:
  (14) $$\tilde{d_{2}}(t)=\frac{1}{I}\sum_{i=1}^{I}E_{1}(r_{1}(t)+\varepsilon_{1}E_{1}(\omega^{i}(t)))$$
(4) The *k*th residue is calculated by (k=2,…,K):
  (15) $$r_{k}(t)=r_{k-1}(t)-\tilde{d_{k}}(t)$$
(5) Decompose realizations $r_{k}(t)+\varepsilon_{k}E_{k}(\omega^{i}(t)),i=1,\ldots ,I,$ until their first EMD mode and define the (*k* + 1)th mode as,
  (16) $$\tilde{d_{\left(k+1\right)}}(t)=\frac{1}{I}\sum_{i=1}^{I}E_{1}(r_{k}(t)+\varepsilon_{k}E_{k}(\omega^{i}(t)))$$
(6) Go to step 4 for next *k*.

Steps 4 to 6 are conducted until the obtained residue is no longer feasible to be decomposed (the residue does not have at least two extrema). The final residue satisfies:

(17) $$R(t)=f(t)-\sum_{k=1}^{K}{\tilde{d}}_{k}(t)$$

with *k* is the number of modes. Therefore, the analyzed signal can be expressed as:

(18) $$f(t)=\sum_{k=1}^{K}{\tilde{d}}_{k}(t)+R(t)$$

Equation (18) makes the proposed decomposition complete and provides an exact reconstruction of the original signal.

According to Equations (14) and (16), the coefficients $\varepsilon_{k}$ can be adjusted to select the signal:noise ratio (SNR) at each stage. For the amplitude of the added noise, Ref. [21] suggested that small-amplitude values are adopted for signals dominated by high-frequency signals, and vice versa. In CEEMDAN, a few hundreds of realizations are done with a fixed SNR for all the stages. This value might depend on the characteristics of the analyzed signal.

#### 2.4.2. Principle of Improved Complementary Ensemble Empirical Mode Decomposition with Adaptive Noises

In the original CEEMDAN [21], the first mode is obtained in the same way as in EEMD. To extract the rest of the modes, a different noise must be added to the current residue. That particular noise is an EMD mode of white noise. For example, to extract the second mode, different copies of $r_{1}(t)+\varepsilon_{1}E_{1}(\omega^{i}(t))$ must decomposed, where *r*_1_ is the first residue. This operation generates a strong overlapping in the scales, and we are focusing in for the first two modes (first one extracted adding white noise and the second one adding $E_{1}(\omega^{i}(t))$. To reduce this overlap, Colominas [22] proposed an improved version of CEEMDAN to make no direct use of white noise but use $E_{k}(\omega^{i}(t))$ to extract the *k*th mode.

In the improved version of CEEMDAN, the operation M(⋅) is denoted as the operator, which produces the local mean of the upper envelope and the lower envelope, and the operation $E_{k}(\cdot )$ is defined which generates the *k*th mode obtained by EMD, and $\omega^{i}$ is denoted as the white noise with N(0,1). The steps of the algorithm are as follows:

(1) The local means of *I* realizations $f^{\left(i\right)}=f+\beta_{0}E_{1}(\omega^{\left(i\right)})$ are calculate by EMD to obtain the first residue
  (19) $$r_{1}=\langle M(f^{\left(i\right)})\rangle$$
(2) At the first stage (*k* = 1), calculate the first mode by
  (20) $${\tilde{d}}_{1}(t)=f(t)-r_{1}(t)$$
(3) The second residue is calculated as the average of local means of the realizations $r_{1}(t)+\beta_{1}E_{2}(\omega^{i}(t))$. The second mode is defined as,
  (21) $${\tilde{d}}_{2}(t)=r_{1}(t)-r_{2}(t)=r_{1}(t)-\langle M(r_{1}(t)+\beta_{1}E_{2}(\omega^{i}(t)))\rangle$$
(4) For *k* = 3, ..., *K*, the *k*th residue is calculated by
  (22) $$r_{k}(t)=\langle M(r_{k-1}(t)+\beta_{k-1}E_{k}(\omega^{i}(t)))\rangle$$
(5) The *k*th mode is calculated by
  (23) $${\tilde{d}}_{k}(t)=r_{k-1}(t)-r_{k}(t)$$
(6) Repeat Steps (4) and (5) to calculate the next $r_{k}(t)$ and ${\tilde{d}}_{k}(t)$.

Constants $\beta_{k-1}=\varepsilon_{k}\mathrm{std}(r_{k})$ (std(*r*) means the standard deviation of *r*) are chosen to obtain a desired SNR between the added noise and the residue to which the noise is added. It should be noticed that, in EEMD, the SNR between the added noise and the residue increases with the order *k*. This is because the energy of the noise in the *k*th residue, *k* > 1, is only a fraction of the energy of the noise added at the beginning of the algorithm. To emulate this behavior, in the algorithm, $\beta_{0}$ is selected in a way that *ε*_0_ is exactly the reciprocal of the desired SNR between the first added noise and the analyzed signal: if the SNR is defined as a quotient of standard deviations, we have $\beta_{0}=\varepsilon_{0}\mathrm{std}(f)/\mathrm{std}(E_{1}(\omega^{\left(i\right)}))$. To obtain noise realizations with a smaller amplitude for the following stages of the decomposition, the added noise is calculated as ($\beta_{k-1}=\varepsilon_{k}\mathrm{std}(r_{k})$, *k* ≥ 1).

#### 2.4.3. Comparisons among These Algorithms

We construct a sample signal *f_sig_*_4_, defined as,

(24) $$\begin{array}{c} f_{sig4}=s_{1}(t)+s_{2}(t)\\ s_{1}(t)=\left\{ \begin{array}{l} 0\\ \sin(65\times 2\pi t)\\ 0 \end{array}\right.\begin{array}{l} 0\leq t\leq 0.5\\ 0.5<t\leq 0.75\\ 0.75<t\leq 1 \end{array}\\ s_{2}(t)=\sin(255\times 2\pi t),0\leq t\leq 1 \end{array}$$

**Figure 19:**
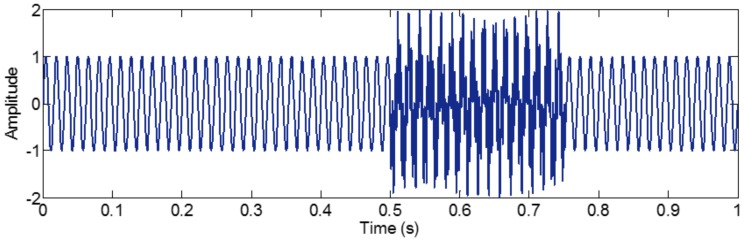
The waveform of the sample signal *f_sig_*_4_.

**Figure 20:**
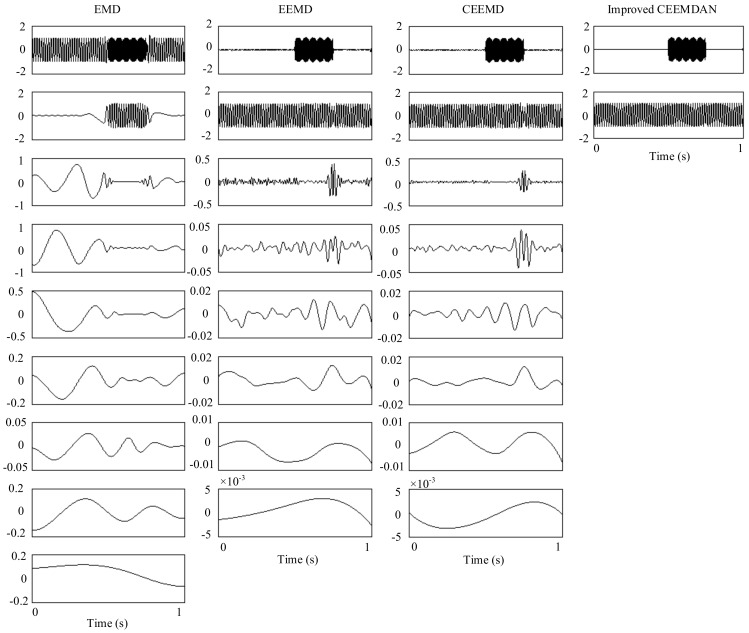
Comparisons among EMD, EEMD, CEEMD and improved CEEMDAN.

The sampling frequency of *f_sig_*_4_ is 1 kHz. The waveform is shown in Figure 19. We employ EMD, EEMD, CEEMD and improved CEEMDAN on *f_sig_*_4_ to obtain comparisons among EMD, EEMD, CEEMD and improved CEEMDAN. The decomposition results are presented in Figure 20. Because the principles of CEEMDAN and improved CEEMDAN are similar in a certain degree, therefore a test for improved CEEMDAN is just done on paper. To quantify the performance of the methods, we set the total number of decompositions as 100, and the amplitude of noise *ε*_0_ as a recommended value of 0.2 for the three noise-assisted EMD variations (EEMD, CEEMD and improved CEEMDAN).

**Figure 21:**
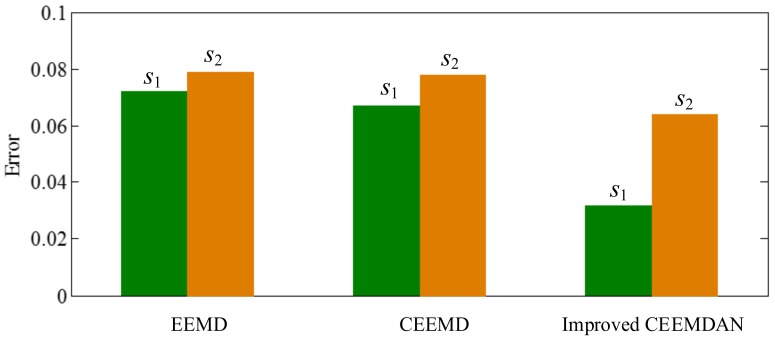
The errors of decomposition results of EEMD, CEEMD and improved CEEMDAN.

As illustrated in Figure 20, for EMD, as mentioned above, in the process of an IMF generation, EMD extracts the component with the highest frequency in every time section, so the component of 255 Hz in time section [0.5 0.75] s is extracted in IMF1 first by EMD (as shown in Figure 20), and mode-mixing occurs. The other noise-assisted EMD variations resolve this problem. However, fewer IMFs are generated in the improved CEEMDAN. To test the accuracy of decomposition result, we define the error with two-norm,

(25) $$err=\frac{{\left\|\mathrm{IMF}i-s_{i}\right\|}_{2}}{{\left\|s_{i}\right\|}_{2}}$$

where IMF *i* represents the *i*th IMF of the decomposition result, and the *s_i_* is the corresponding component of the original signal. The errors of decomposition results of EEMD, CEEMD and improved CEEMDAN are shown in Figure 21. As can be seen in Figure 21, the result errors of improved CEEMDAN also are less than that of EEMD and CEEMD.

### 2.5. Applications and Other Improvement Works of Empirical Mode Decomposition

EMD can decompose a signal into several narrow-band components, which introduces the attractive feature of robustness in the presence of non-linear and non-stationary data. Therefore, a great number of investigations of theory and application have been done for EMD [23,24,25,26,27,28,29,30,31,32,33,34,35,36,37,38,39,40,41,42,43,44,45,46,47,48,49,50,51,52,53,54,55,56,57,58]. Ref. [6] reviewed the essential problems in improvement work and application. Ref. [23] reviewed recent mathematical progress on constructing a large bank of basic functions, establishing a fast adaptive decomposition algorithm, piecewise linear spectral sequences and a Bedrosian identity. Ref. [24] reviewed works on new stopping criteria and an online version of the algorithm. Ref. [25] discuss the way EMD behaves in stochastic situations involving broadband noise. In addition, the references above are all valuable for understanding and employing EMD. In this paper, we summarize some works that were published recently in mechanical engineering, consisting of three parts that are currently popular research issues, i.e., current applications of parameter identification of the mechanical system assisted by algorithms deriving from EMD, techniques applied to process decomposition results obtained by using EMD, or improved methods deriving from EMD about fault diagnosis and other improvement works of EMD about fault diagnosis. The applications of EMD above are based on precisely extracting targeted mono-components, which can be taken as a kind of precise filter. Another application of EMD can be taken as rough filter. Extracting fault signals can be taken as a typical application of rough filter. In addition, the aim focuses on highlighting some quantitative evaluation parameters of fault information.

For precise filter operation, as time–frequency transformations generally offer useful insight into the dynamics of non-linear systems, EMD was widely employed to make parameter identification of mechanical systems more achievable. Yang [26,27] used EMD to isolate different modal responses from free vibrations, and then HT was applied to the instantaneous amplitude and phase angle time histories, which provide a basis for identifying the natural frequency and damping ratio of multi-degree-of-freedom linear systems. Khan [28] employed EEMD and Pareto technique to extract valuable components. After that, Recursive Stochastic Subspace Identification was employed to carry out the continuous modal parameter identification of the cable-stayed bridge. Pai [29] took time-varying amplitude and frequency of the first component extracted by EMD and HT as indicators for pinpointing times and locations of impulsive external loads to obtain extracting characteristics of non-linear systems and intermittent transient responses. Lee [30] developed a time-domain non-linear system identification technique based on EMD. Eriten [31] applied EMD to decompose a given measured velocity signal in terms of IMFs that provided information about the modal content of the signal, which provided a foundation for a non-linear system identification of frictional effects in a beam with a bolted joint connection., presenting a novel method based on Hilbert Huang Transform (HHT), combined by EMD and HT, for analyzing the non-linear and non-stationary Aerial Planting Projectile flight data signal. Chen [33] performed non-linear system identification on the acceleration signals that were experimentally measured at ten almost evenly spaced positions along a cantilever beam undergoing vibro-impacts between two rigid stops with clearances. In addition, EMD was used to obtain sets of intrinsic modal oscillators governing the vibro-impact dynamics at different time scales. Poon [34] attempted to use EMD to identify properties of non-linear elastic multi-degree-of-freedom structures. The IMFs obtained by EMD were used in the context of the non-linear normal mode method to estimate the properties of the non-linear elastic structure. Pai [35] presented a signal-processing methodology based on EMD and a new conjugate-pair decomposition method for characterization of non-linear normal modes and parametric identification of non-linear multiple-degree-of-freedom dynamical systems.

To make fault diagnosis more convenient, some scholars employed some techniques on decomposition results. Bustos [36] proposed an efficient methodology based on EMD, which provided a set of parameters for the fast identification of the operating state of a high-speed train. Van [37] presented a novel two-stage feature selection, hybrid distance evaluation technique-particle swarm optimization to select the superior combining feature subset that discriminates well among classes. On this basis, a comparison among three types of popular classifiers—*K*-nearest neighbors, probabilistic neural network and support-vector machine—was made to establish the sensitivity of each classifier corresponding to the irrelevant and redundant features, and the curse of dimensionality. Wang [38] applied sample entropy to characterize the complexity of IMFs obtained by using CEEMD in different time scales. Then, a random forest classifier was untiled for identification and classification of fault modes of centrifugal pumps. Ali [39] used an artificial neural network to classify bearings defects, and a mathematical analysis to select the most significant IMFs. Zhang [40] utilized support vector machines optimized by inter-cluster distance in the feature space to classify the fault type. The permutation entropy values of the first few IMFs obtained by using EEMD were taken to reveal the multi-scale intrinsic characteristics of signals. Georgoulas [41] extracted fault features by using HHT, and then trained a hybrid ensemble detector to obtain detection of any deviation from the normal condition. Further, Georgoulas [42] employed hidden Markov models to automatically identify fault, and the inputs were feather parameters obtained by using complex EMD and HT. Meng [50] also employed a hidden Markov model classifier for malfunction recognition, in which the instantaneous energy distribution of signals were taken as the inputs. Zhao [45] quantitatively evaluated the complexity of the IMFs to obtain quantitative diagnosis of a spall-like fault of a rolling element bearing. Djebala [46] used an optimized wavelet multi-resolution analysis to analyze envelope spectrums of optimal IMFs and highlight the fault characteristic frequency. Bi-spectrums, a third-order statistic, which helps to identify phase coupling effects of IMFs were used to detect outer race bearing defects by Saidi [47]. Le [48] employed the radial basis function neural network based on chemical reaction optimization algorithms to identify the work condition of the gear, in which, the energy features extracted from valuable IMFs were taken as the inputs. Wang [49] applied independent component analysis (ICA) technique on IMFs that contained information of compound faults to effectively separate component fault features.

Apart from the mentioned techniques, i.e., EEMD, CEEMD, CEEMDAN and improved CEEMDAN, some other improved versions of EMD were also proposed, which also have superior qualities to EMD. Zheng [51] proposed an adaptive data-driven analysis approach called generalized empirical mode decomposition (GEMD), in which different baselines were firstly defined and separately subtracted from the original data, and then different pre-generated intrinsic mode functions were obtained. Next, the pre-generated intrinsic mode function was subtracted from the original signal. A demodulating method called empirical envelope demodulation (EED) was introduced. Results revealed that the method consisting of GEMD and EED performed better in restraining the end effect, gaining a better frequency resolution and more accurate time frequency distribution. Zheng [53] also presented another improved version of EMD called partly ensemble EMD (PEEMD) to resolve the mode-mixing problem. In PEEMD, after the intermittency or noise signal was obtained in an ensemble way and was detected by permutation entropy, the residual signal was decomposed directly by using EMD. Similarly to Ref. [49], Jiang [54] put forward an algorithm called improved EEMD with multiwavelet packet, in which multiwavelet packet was used as the pre-filter to improve EEMD decomposition results. The result showed that the method can keep weak multi-fault characteristic components. Table 1 is designed to make this section more readable.

## 3. Empirical Wavelet Transform

In 2013, Gilles [59] proposed a novel adaptive decomposition entitled EWT, which combines merits of EMD and WT. EWT utilizes the Meyer wavelet siding along the time axis to conduct reconstruction instead of the orthogonal basis of sine wave, so the local characteristics of signals tend to be more accurately described than Fourier transform (FT). Moreover, in contrast with adaptive decomposition algorithms such as EMD, the basic function of Meyer wavelet generates in the calculation process of the inner product between the Fourier spectrum of signals and the Fourier spectrum of the Meyer wavelet; therefore, the frequency resolution of EWT depends on the frequency resolution of FT posing a promising frequency resolution, which can be easily deduced from Heisenberg’s uncertainty principle.

### 3.1. Principle of Empirical Wavelet Transform

**Figure 22:**
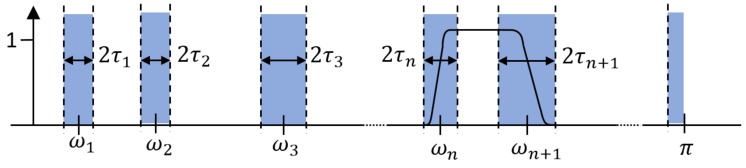
Segmenting Fourier spectrum into *N* contiguous segments.

The principle can be found in Ref. [59], and we briefly explain the theory of EWT in the paper. There is an assumption in EWT that the Fourier support is segmented into contiguous segments. Segmenting the Fourier spectrum generates the limits between each segment (where $\omega_{0}=0$ and $\omega_{N}=\pi$, the total number of segmenting section is *N*). $\Lambda_{n}=[\omega_{n-1},\omega_{n}]$ represents each segment. It is defined that a transition phase *T_n_* centers around each $\omega_{N}$. The empirical wavelets act as bandpass filters on each $\Lambda_{n}$, as shown in Figure 22. When ∀n>0, Equations (26) and (27) define the empirical scaling function and the empirical wavelets, respectively.

(26) $${\hat{\varphi }}_{n}(\omega )=\left\{ \begin{array}{ccc} 1 & , & \left|\omega \right|\leq \omega_{n}-\tau_{n}\\ \cos\left[\frac{\pi }{2}\beta (\frac{1}{2\tau_{n}}(\left|\omega \right|-\omega_{n}+\tau_{n})\right] & , & \omega_{n}-\tau_{n}\leq \left|\omega \right|\leq \omega_{n}+\tau_{n}\\ 0 & , & \mathrm{otherwise} \end{array}\right.$$

and

(27) $$\begin{array}{c} {\hat{\psi }}_{n}(\omega )=\left\{ \begin{array}{lll} 1 & , & \omega_{n}+\tau_{n}\leq \left|\omega \right|\leq \omega_{n}{}_{+1}-\tau_{n+1}\\ \cos\left[\frac{\pi }{2}\beta (\frac{1}{2\tau_{n}}(\left|\omega \right|-\omega_{n+1}+\tau_{n+1}))\right] & , & \omega_{n+1}-\tau_{n+1}\leq \left|\omega \right|\leq \omega_{n+1}+\tau_{n+1}\\ \sin\left[\frac{\pi }{2}\beta (\frac{1}{2\tau_{n}}(\left|\omega \right|-\omega_{n}+\tau_{n}))\right] & , & \omega_{n}-\tau_{n}\leq \left|\omega \right|\leq \omega_{n}+\tau_{n}\;\\ 0 & , & \mathrm{otherwise} \end{array}\right. \end{array}$$

The function β(x) is an arbitrary function $C^{k}(\left[0,1\right])$ that subjects to

(28) $$\left\{ \begin{array}{lll} \beta (x)=0 & , & x=0\\ \beta (x)+\beta (1-x)=1 & , & \forall x\in (0,1)\\ \beta (x)=1 & , & x=1 \end{array}\right.$$

To obtain $\tau_{n}$, proportional is chosen to $\omega_{n}:\tau_{n}=\gamma \omega_{n}$ where 0<γ<1. Consequently, ∀n>0, Equations (26) and (27) can simplify to Equations (29) and (30),

(29) $${\hat{\varphi }}_{n}(\omega )=\left\{ \begin{array}{lll} 1 & , & \left|\omega \right|\leq (1-\gamma )\omega_{n}\\ \cos\left[\frac{\pi }{2}\beta (\frac{1}{2\gamma \omega_{n}}(\left|\omega \right|-(1-\omega )\omega_{n}))\right] & , & (1-\gamma )\omega_{n}\leq \left|\omega \right|\leq (1+\gamma )\omega_{n}\\ 0 & , & \mathrm{otherwise} \end{array}\right.$$

and

(30) $$\begin{array}{c} {\hat{\psi }}_{n}(\omega )=\left\{ \begin{array}{lll} 1 & , & (1+\gamma )\omega_{n}\leq \left|\omega \right|\leq (1-\gamma )\omega_{n+1}\\ \cos\left[\frac{\pi }{2}\beta (\frac{1}{2\gamma \omega_{n}}(\left|\omega \right|-(1-\gamma )\omega_{n+1}))\right] & , & (1-\gamma )\omega_{n+1}\leq \left|\omega \right|\leq (1+\gamma )\omega_{n+1}\\ \sin\left[\frac{\pi }{2}\beta (\frac{1}{2\gamma \omega_{n}}(\left|\omega \right|-(1-\gamma )\omega_{n}))\right] & , & (1-\gamma )\omega_{n}\leq \left|\omega \right|\leq (1+\gamma )\omega_{n}\\ 0 & , & \mathrm{otherwise} \end{array}\right. \end{array}$$

To get the boundaries *ω_n_*, we can segment the Fourier spectrum of signal on the basis of local maxima. The parameter *γ* can be set as value in internal [0, γ0) (Then the set $\left\{\varphi_{1}(t),\text{ \{}\psi_{n}(t{)\text{\}}}_{n=1}^{N}\right\}$ is an orthonormal basis of $L^{2}(\mathbb{R})$ and γ_0_ is calculated by,

(31) $$\gamma_{0}=\arg\min(\frac{\omega_{n+1}-\omega_{n}}{\omega_{n+1}+\omega_{n}})$$

$W_{f}^{\varepsilon }(n,t)$ is defined as the Empirical Wavelet Transform. The detail coefficients are given by the inner product with the empirical wavelets:

(32) $$W_{f}^{\varepsilon }(n,t)=\langle f,\psi_{n}\rangle =\int f(\tau )\bar{\psi_{n}(\tau -t)}d\tau ={\left(\hat{f}(\omega )\bar{{\hat{\psi }}_{n}(\omega )}\right)}^{\vee }$$

and the approximation coefficients $W_{f}^{\varepsilon }(0,t)$ is adopted to denote them) by the inner product with the scaling function:

(33) $$W_{f}^{\varepsilon }(0,t)=\langle f,\varphi_{1}\rangle =\int f(\tau )\bar{\varphi_{1}(\tau -t)}d\tau ={\left(\hat{f}(\omega )\bar{{\hat{\varphi }}_{1}(\omega )}\right)}^{\vee }$$

where ${\hat{\varphi }}_{1}(\omega )$ and ${\hat{\psi }}_{n}(\omega )$ are defined by Equations (27) and (28), respectively. The reconstruction is obtained by

(34) $$\begin{array}{r} f(t)=f_{0}(t)+\sum_{n=1}^{N}f_{n}(t)=W_{f}^{\varepsilon }(0,t)*\varphi_{1}(t)+\sum_{n=1}^{N}W_{f}^{\varepsilon }(n,t)*\psi_{n}(t)\\ \;=(\hat{W_{f}^{\varepsilon }}(0,\omega )\cdot \varphi_{1}(\omega )+\sum_{n=1}^{N}\hat{W_{f}^{\varepsilon }}(n,\omega )\cdot \psi_{n}(\omega ){)}^{\vee } \end{array}$$

where, * denotes the convolution operators.

### 3.2. Advantage of Empirical Wavelet Transform

As mentioned above, the frequency resolution of algorithms deriving from EMD is a basic limitation. When the ratio between a relatively low frequency and a relatively high frequency is larger than 0.75, the two components of a signal cannot be separated. This limitation can be broken by EWT, as its frequency resolution depends on Fourier spectrum posing a promising frequency resolution. To illustrate it, a sample signal fsig5 is employed.

(35) $$\begin{array}{c} f_{sig5}=s_{1}(t)+s_{2}(t)+s_{3}(t)\\ s_{1}(t)=\sin(50\times 2\pi t),0\leq t\leq 1\\ s_{2}(t)=\sin(600\times 2\pi t),0\leq t\leq 1\\ s_{3}(t)=\sin(800\times 2\pi t),0\leq t\leq 1 \end{array}$$

**Figure 23:**
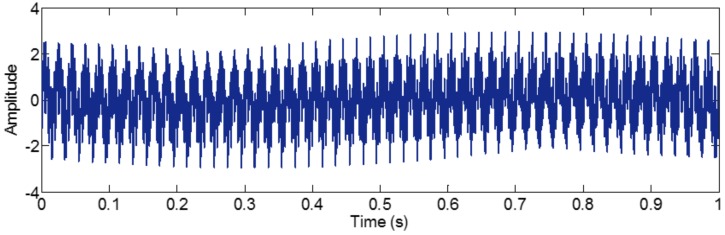
The waveform of the sample signal *f_sig_*_5_.

**Figure 24:**
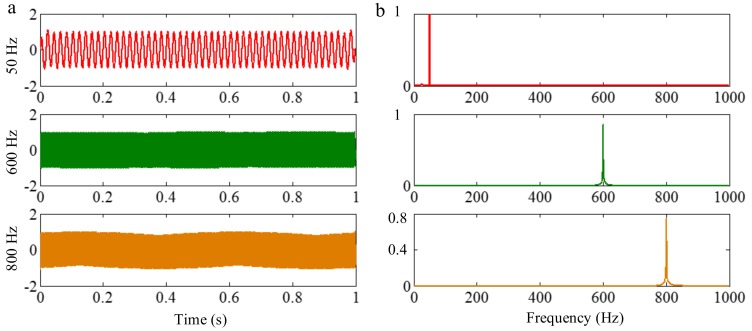
The waveforms of EWT result of *f_sig_*_5_ and the corresponding Fourier spectrums: (**a**) waveforms and (**b**) Fourier spectrums.

**Figure 25:**
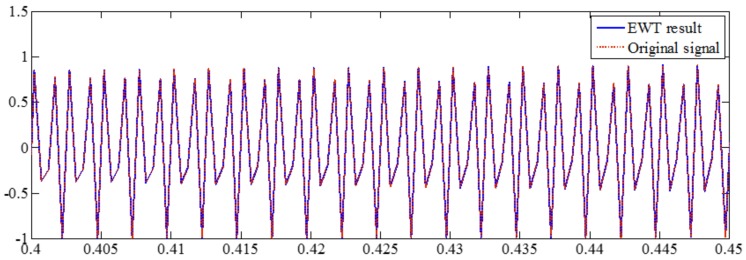
The EWT result and the original signal of 800 Hz within [0.4 0.45] s.

The sampling frequency is 2 kHz, as shown in Figure 23. EWT successfully separate components of 50, 600 and 800 Hz, as shown in Figure 24. In Section 2.1.3 and Section 2.2.4, it has been shown that the sampling frequency can influence the decomposition result by using algorithms deriving from EMD. When the sampling frequency is not sufficient enough, the errors of extreme location can result in error of decomposition result. However, EWT is immune to this within limitation of Shannon’s sampling theorem. To illustrate this, we show the EWT result and the original signal of 800 Hz within [0.4 0.45] s in Figure 25. As shown in Figure 25, the EWT result almost overlaps with the original signal, which indicates a high accuracy of EWT.

### 3.3. Disadvantage of Empirical Wavelet Transform

#### 3.3.1. Limitation of Segmenting Fourier Spectrum

Since empirical wavelets are generated by segmenting Fourier spectrum, when different components of a signal cannot be separated in Fourier spectrum, decomposition results of EWT will not be correct. To illustrate this, we construct a sample signal *f_sig_*_6_

(36) $$\begin{array}{c} f_{\mathrm{sig}6}(t)=s_{1}(t)+s_{2}(t)+s_{3}(t),0\leq t\leq 1\\ s_{1}=\left\{ \begin{array}{l} 4t\sin[(50+100t)\cdot 2\pi \cdot t]\\ -\frac{4}{3}(t-1)\sin[(50+100t)\cdot 2\pi \cdot t] \end{array}\right.\begin{array}{l} ,\;0\leq t<0.25\\ ,\;0.25\leq t<1 \end{array}\\ s_{2}=\left\{ \begin{array}{l} 2t\sin[(100+200t)2\pi \cdot t]\\ -2(t-1)\sin[(100+200t)\cdot 2\pi \cdot t] \end{array}\right.\begin{array}{l} ,\;0\leq t<0.5\\ ,\;0.5\leq t<1 \end{array}\\ s_{3}=\left\{ \begin{array}{l} \frac{4}{3}t\sin[(200+400t)\cdot 2\pi \cdot t]\\ -4(t-1)\sin[(200+400t)\cdot 2\pi \cdot t] \end{array}\right.\begin{array}{l} ,\;0\leq t<0.75\\ ,\;0.75\leq t<1 \end{array} \end{array}$$

**Figure 26:**
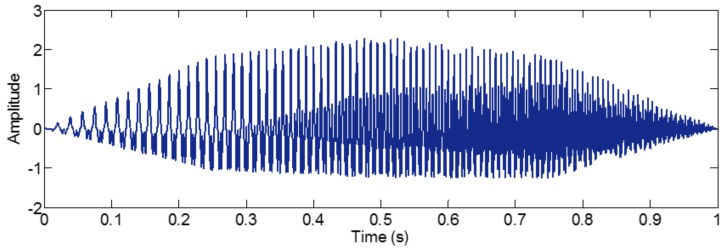
The waveform of the sample signal *f_sig_*_6_.

**Figure 27:**
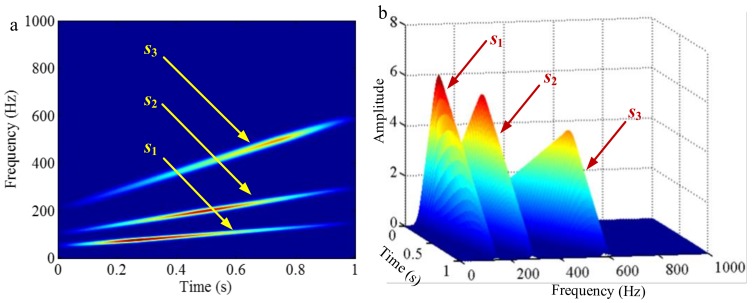
The STFT of the sample signal *f_sig_*_6_: (**a**) 2D figure and (**b**) 3D figure.

**Figure 28:**
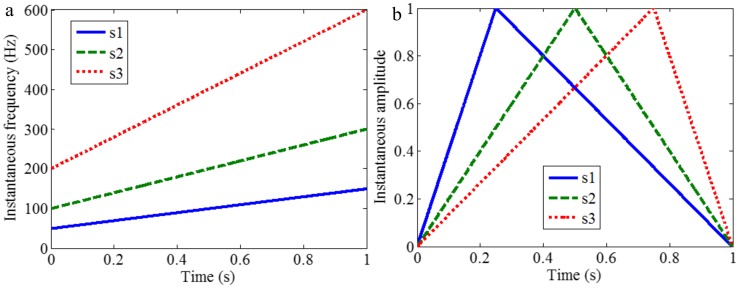
The IF and IA of the sample signal *f_sig_*_6_: (**a**) IF and (**b**) IA.

**Figure 29:**
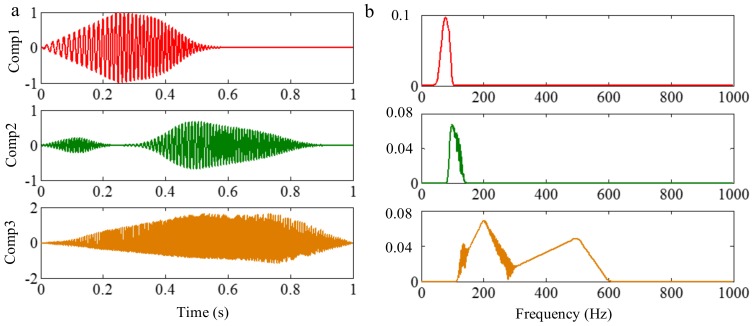
The waveforms of EWT result of *f_sig_*_6_ and the corresponding Fourier spectrums: (**a**) waveforms and (**b**) Fourier spectrums.

The sampling frequency is 2 kHz. The waveform of *f_sig_*_6_ is shown in Figure 26, and the corresponding STFT representation is shown in Figure 27. The IF and IA of the sample signal *f_sig_*_6_ are shown in Figure 28. We employ EWT to process the sample signal *f_sig_*_6_, and the result is shown in Figure 29. It is easy to establish that the EWT of the sample signal *f_sig_*_6_ is unsuccessful. Moreover, there are some negative factors for finding boundaries of different mono-components, for example white noise that can introduce redundant extremes in the Fourier spectrum, which are essential for establishing boundaries of mono-components. Therefore, further work can be done to eliminate such negative influences.

#### 3.3.2. Limitation of Selection of Detection Method of Boundary

**Figure 30:**
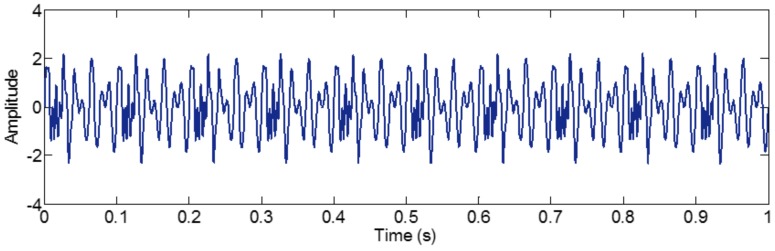
The waveform of the sample signal *f_sig_*_7_.

**Figure 31:**
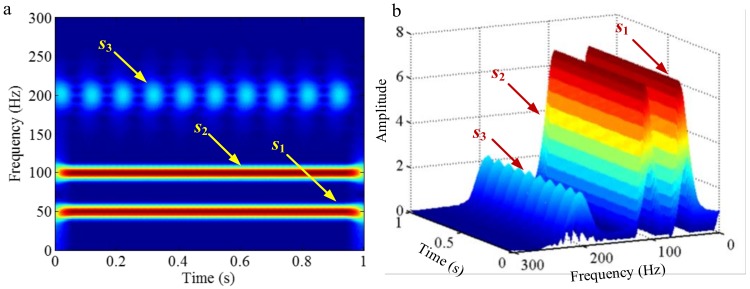
The STFT of the sample signal *f_sig_*_7_: (**a**) 2D figure and (**b**) 3D figure.

**Figure 32:**
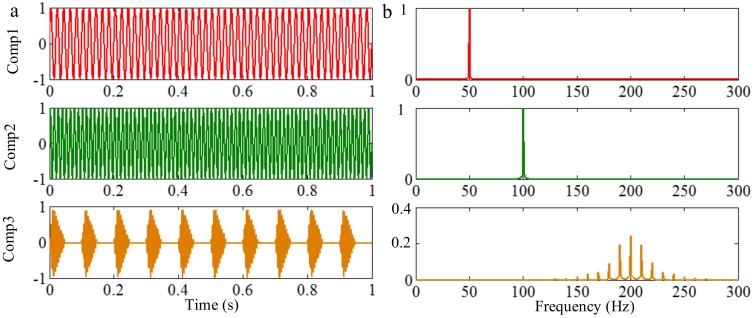
Each component of the sample signal *f_sig_*_7_: (**a**) the waveform and (**b**) the Fourier spectrum.

**Figure 33:**
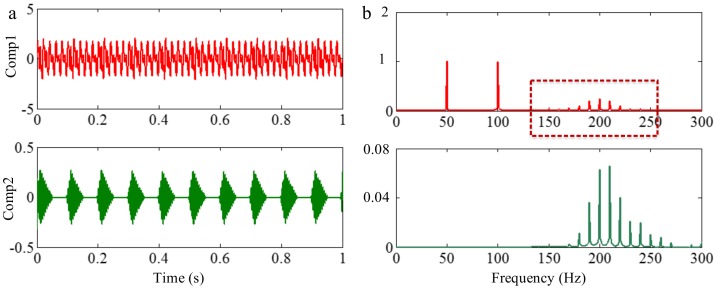
The waveforms of EWT result of *f_sig_*_6_ and the corresponding Fourier spectrums: (**a**) waveforms and (**b**) Fourier spectrums. The parameters used in processed code are as follows: params.detect is set as ‘adaptivereg’, params.typeDetect is set as ‘otsu’.

Successful decomposition of multi-component signals depends on segmenting the corresponding Fourier spectrum by using EWT. To improve the adaptivity of EWT, several detection methods of boundary are available in the code of EWT [60]. The same computation results may be obtained when the Fourier spectrum of the signals are simple. However, when the spectrum is complicated, a suitable method should be selected to improve decomposition result. To illustrate this, we construct a sample signal *f_sig_*_7_, as shown in Figure 30, and the corresponding STFT representation is shown in Figure 31. Each component of the sample signal *f_sig_*_7_ is shown in Figure 32. It consists of components of 50, 100 and 200 Hz, and the component of 200 Hz is amplitude-modulated. They are defined as components 1–3. The sampling frequency is 2 kHz. As presented in Figure 32b, the Fourier spectrum of component 3 is complicated. We process *f_sig_*_7_ by EWT, setting parameters used in processed code as follows, params.detect is set as “adaptivereg”, params.typeDetect is set as “otsu”. As shown in Figure 33, it is clear that the decomposition result of *f_sig_*_7_ is not promising. Therefore, when the signal is non-stationary, the Fourier spectrum tends to become complicated, and EWT will easily fail when doing mode separation. Though we can obtain a successful result by changing the detection method of the boundary, robustness of EWT is not promising. Researchers should pay attention to these characteristics to guarantee that the employment of EWT is correct.

### 3.4. Application and Improvement Works of Empirical Wavelet Transform

As illustrated above, EWT can separate different mono-components that do not overlap in the Fourier spectrum. Sometimes, we can employ this method to extract specific mono-components to a high accuracy. Yuan [61] presented a technique that combined the second-order blind identification method with the EWT to delineate closely spaced frequencies. In addition, EWT operated on the modal responses estimated by the SOBI and yielded the closely spaced natural frequencies. Hu [62] proposed a hybrid model that was composed of EWT, partial auto-correlation function and Gaussian process regression method for short-term wind speed prediction. In this approach, EWT was employed to extract meaningful information from a wind speed series by designing an appropriate wavelet filter bank. Reddy [63] applied EWT to extract the actual fundamental frequency component and disturbance components from distorted signals. In addition, then, time-varying power quality indices for accurate assessment of Power Quality Disturbances were estimated. Thirumala [64,65] proposed two different algorithms for the estimation of power-quality indices based on EWT. The results confirmed that EWT efficiently extracts the mono-component signals from the actual distorted signal and thereby accurately estimates the power quality indices. Li [66] proposed a novel approach for capturing the instantaneous pitch that may reveal some innate character of the speech, and EWT was employed to pick out the mode containing the pitch. Liu [67] presented an algorithm combining EWT, HT and short time Fourier transform obtain the TFD of ultrasonic testing waves. The different wave packages were isolated using EWT.

In some situations, some residual noise is tolerable under the premise that the specific signal feature is strong enough, for example in fault diagnosis of machines, which is a popular research issue at present. In addition, EWT also can remove other unvalued components. Therefore, EWT and its improved version have been successfully employed in fault diagnosis of machines [68,69,70,71,72,73,74,75,76,77,78,79,80,81,82].

Since the main idea of EWT is defining a bank of wavelet filters based on the “well-chosen” Fourier supports, establishing targeted boundaries of the filters is key to extracting fault components from raw signals. To guarantee the obtaining of correct boundaries, some scholars also conducted investigations on the issue. Gilles [68] proposed a parameterless scale-space approach, which is easy to implement, is fast, and does not require any parameter, to find meaningful modes in histograms-application spectrum segmentation. The algorithm is based on the behavior of local minima in a scale-space representation, and the detection of such meaningful modes is the equivalent to a two-class clustering problem on the length of minima scale-space curves. Based on this method, Zheng [69] presented an improved version of EWT called adaptive parameterless EWT, in which the adaptive segmentation of Fourier spectrum led to the adaptive separation of empirical wavelets. In Ref. [70], the peak characteristic of autocorrelation function was used to judge the periodicity of each signal, and the most obvious signal was taken as the characteristic signal. An iteration decomposition of trend was presented. Kedadouche [71] presented another method of segmentation of the Fourier spectrum. The aim of the method was to separate different portions of the spectrum which were centered on a specific frequency, which presented the highest amplitude. To remove more useless components, the decomposition target was not the raw testing signal, but Combined Mode Function obtained by combining neighboring IMFs obtained from the EMD of the raw signal. Moreover, Kedadouche [72] also pointed out that EWT acts like a filter bank and employed operational modal analysis to define the support boundaries of the filter, and the algorithm was called operational modal analysis-empirical wavelet transform, which was better than the original version of EWT presented in Ref. [49] at decomposing multiple-component signals. To avoid the inaccurate segmentation of Fourier spectrum resulted from noises, Chen [73] conducted a de-noising operation by using wavelet spatial neighboring coefficient de-noising with a data-driven threshold. The result indicated that the technique was effective on weak fault and compound fault diagnosis. Pan [74] proposed a data-driven adaptive Fourier spectrum segment method for mechanical fault identification. In this technique, the inner product was first calculated between the Fourier spectrum of the analyzed signal and the Gaussian function for scale representation, and then, local minima of the scale representation were detected to obtain the adaptive spectrum segment. Hu [75] modified the segmentation algorithm by using the envelope approach based on the order statistics filter and applying criteria to pick out useful peaks. The proposed method obtained a perfect segmentation in decomposing noisy and non-stationary signals.

In some references, EWT or its improved version is firstly employed to decompose signals to obtain valuable components that carry defect information. Then, another technique is used to process these valuable components to obtain detection of fault. Specifically, Huang [76] used one-class support vector machine to value components to achieve fault detection of high-voltage circuit breakers. Following HHT, HT is used to obtain IFs and IAs of valuable components to obtain early detection of tooth-crack damage in a gearbox [77,78]. In Ref. [79], each single fault frequency was incorporated into a duffing oscillator to establish its corresponding fault isolator, and the single faults were identified one by one from the empirical modes by directly observing the chaotic motion from the Poincar mapping of the isolator outputs. Zheng [69] presented an improved version of HT called quadrature derivative-based normalized Hilbert transform to process valuable components, and the proposed method could effectively fulfill the fault diagnosis of rotor rubbing.

## 4. Variational Mode Decomposition

As mentioned above, the selection of detection method of boundary is an inconvenience of EWT. Therefore, an adaptive decomposition algorithm without this operation may be more welcome. In 2014, Dragomiretskiy [83] proposed VMD, determining the relevant bands adaptively. The method can estimate the corresponding modes concurrently, thus perfectly balancing errors between them to obtain separation of different components from signals.

### 4.1. Principle of Variational Mode Decomposition

In VMD, Wiener filtering, the HT and heterodyne demodulation are combined, and an alternate direction method of multipliers (ADMM) is employed to obtain decomposing modes. The decomposed modes are localized on central frequencies. The bandwidth of a decomposed mode is generated in the following ways [83]:

(1) HT is employed to estimate the one-sided frequency spectrum of a real signal using an analytic representation.
(2) The modulation properties are utilized to obtain the shift of the frequency spectrum of the mode is shifted to the estimated base-band frequencies.
(3) The H^1^ Gaussian smoothness of the demodulated signal is applied to estimate the bandwidth.

VMD represents a signal *f*(*t*) with a set of components called modes $\varphi_{k}(t)$ localized on the center frequency $\omega_{k}$. ADDM is employed to resolve the constrained variational optimization problem, which can be expressed by

(37) $$\begin{array}{c} \underset{\left\{\varphi_{k}\},\{\omega_{t}\right\}}{\min}\{\sum_{k=1}^{K}{\left\|\partial_{t}[(\delta (t)+\frac{j}{\pi t})*\varphi_{k}(t)]e^{-j\omega_{k}t}\right\|}_{2}^{2}\},\\ \mathrm{Subject}\;\mathrm{to}\;f(t)=\sum_{k=1}^{K}\varphi_{k}(t) \end{array}$$

where *δ* denotes the Dirac distribution, and * and ∂ denote the convolution and partial differential operators, respectively. Equation (37) can be addressed by introducing a quadratic penalty and Lagrangian multipliers. The augmented Lagrangian is given as follows:

(38) $$\begin{array}{l} L(\{\varphi_{k}\},\{\omega_{k}\},\lambda )=\alpha \sum_{k=1}^{K}{\left\|\partial_{t}[(\delta (t)+\frac{j}{\pi t})*\varphi_{k}(t)]e^{-j\omega_{k}t}\right\|}_{2}^{2}+\\ {\left\|f(t)-\sum_{k=1}^{K}\varphi_{k}(t)\right\|}_{2}^{2}+\langle \lambda (t),f(t)-\sum_{k=1}^{K}\varphi_{k}(t)\rangle . \end{array}$$

The modes $\varphi_{k}(t)$ in the frequency domain are estimated using ADDM in the form of the Wiener filter structure as follows:

(39) $${\hat{\varphi }}_{k}(\omega )=\frac{\hat{f}(\omega )-\sum_{i\neq k}{\hat{\varphi }}_{i}(\omega )+(\hat{\lambda }(\omega )/2)}{1+2\alpha {\left(\omega -\omega_{k}\right)}^{2}}$$

where ${\hat{\varphi }}_{k}(\omega )$, $\hat{f}(\omega )$, ${\hat{\varphi }}_{i}(\omega )$ and $\hat{\lambda }(\omega )$ are the FT of the components. Finally, the modes in the time domain are obtained computing the inverse FT of the filtered signal, and the center frequencies are estimated by

(40) $$\omega_{k}=\frac{\int_{0}^{\infty }\omega {\left|{\hat{\varphi }}_{i}(\omega )\right|}^{2}d\omega }{\int_{0}^{\infty }{\left|{\hat{\varphi }}_{i}(\omega )\right|}^{2}d\omega }$$

Thus, it is feasible to analyze the sub-components of a signal with the modes having localized center frequency properties. In this paper, we briefly illustrate the principle of VMD. The detail of calculation process can be found in Ref. [83].

### 4.2. Advantage of Variational Mode Decomposition

**Figure 34:**
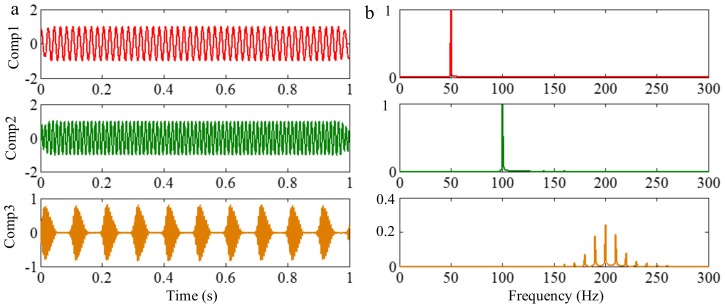
The decomposition result of *f_sig_*_7_: (**a**) the waveform and (**b**) the Fourier spectrum.

As mentioned above, VMD can determine the relevant bands adaptively. To invalidate this characteristic, we employ VMD to process the sample signal *f_sig_*_7_. Figure 34 shows the decomposition result of *f_sig_*_7_. As presented in Figure 34, the three sub-components of *f_sig_*_7_ are separated by VMD. Comparing Figure 33 with Figure 34, it is easy to learn that the decomposition result obtaining be VMD is of a high accuracy, except for some end errors. So the end effect is also a research issue that needs to be resolved. Moreover, another advantage should be noted for VMD. The resulting optimization scheme is very simple and fast. In VMD, the narrow-band Wiener filter that corresponds to the current estimate of the mode’s center-frequency is applied to the signal estimation residual of all other modes, which generates each mode in iteratively updating directly Fourier spectrum. The center frequency is re-estimated as the center of gravity of the mode’s power spectrum. The computation intensity of these processes is low.

### 4.3. Disadvantage of Variational Mode Decomposition

**Figure 35:**
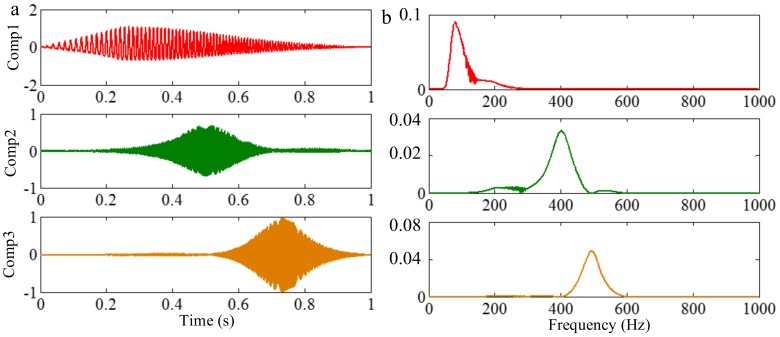
The decomposition result of *f_sig_*_6_ by using VMD: (**a**) the waveform and (**b**) the Fourier spectrum.

**Figure 36:**
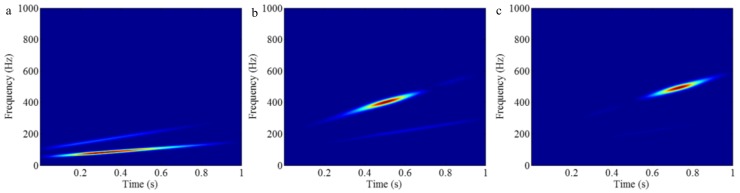
The STFT representations of the Comps 1–3: (**a**) Comp 1; (**b**) Comp 2 and (**c**) Comp 3.

VMD segments the Fourier spectrum to obtain separating different components of a signal. Similar to EWT, therefore, this method also suffers from the limitation of the Fourier spectrum, i.e., when different components cannot be separated in the Fourier spectrum, they cannot be separated by VMD. There, VMD is also employed to process *f_sig_*_6_, and the result is shown in Figure 35, and the STFT representations of the Comps 1–3 are shown in Figure 36. As shown in Figure 28a, the frequencies of components 1–3 are, respectively, in intervals [50 150], [100 300] and [200 600]. However, the frequencies of components 1–3 are in intervals [50 250], [150 600] and [400 600], as shown in Figure 35b. So the VMD of *f_sig_*_6_ is unsuccessful, which can be further verified by comparing Figure 27 with Figure 36.

**Figure 37:**
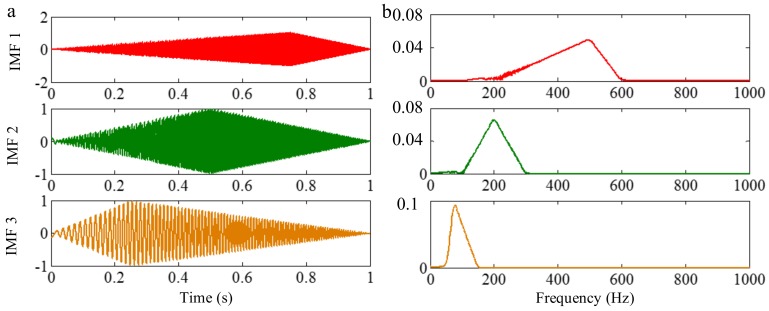
The decomposition result of *f_sig_*_6_ by using EMD: (**a**) the waveform and (**b**) the Fourier spectrum.

**Figure 38:**
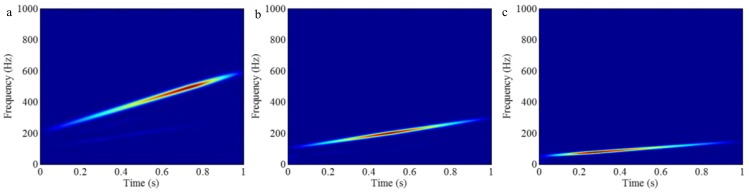
The STFT representations of the IMFs 1–3: (**a**) IMF 1; (**b**) IMF 2; and (**c**) IMF 3.

However, a promising decomposition result can be obtained by using EMD. Figure 37 shows the decomposition result of *f_sig_*_6_ by using EMD, and the corresponding STFT representations are shown in Figure 38 (it should be noted that IMFs 1–3 correspond to components 3–1 of *f_sig_*_6_). As presented in Figure 37 and Figure 38, EMD successfully separates the three components of *f_sig_*_6_. Therefore, EMD does not suffer this limitation of the Fourier spectrum.

### 4.4. Application and Improvement Works of Variational Mode Decomposition

As illustrated above, in VMD, separating different modes of a signal is translated into a constrained variational optimization problem. Therefore VMD allows adaptive decomposition of the signal into various modes by identifying a compact frequency support around its central frequency. Similar to EWT, the method can be employed to accurately extract specific mono-components from raw signals, and then some parameter estimations can be obtained. Upadhyay proposed approaches to obtain the instantaneous detection of voiced/non-voiced regions in the speech signals [84] and determine instantaneous fundamental frequency [85] of speech signals based on VMD. Yin [86] presented a microwave propagating mode extraction algorithm for microwave waveguide using VMD. A coated steel defect detection experiment was conducted using an X-band open-ended rectangular waveguide to evaluate the efficacy of VMD. In addition, for two samples, the VMD results could accurately identify the defects. Gao [87] proposed an online evaluation of metal burn degrees based on acoustic emission and VMD, and VMD was applied to extract the main frequency of AE burn signals. To resolve the problem that the features of ship-radiated noise were difficult to extract and were inaccurate, Li [88] presented a method based on VMD, multi-scale permutation entropy) and a support vector machine to extract the features of ship-radiated noise.

Similar to EWT, as the fault diagnosis of machinery is a popular issue of dynamic analysis, a great amount of research regarding application and improvement has been undertaken for VMD in this domain [89,90,91,92,93,94,95,96,97,98,99,100,101,102,103,104,105,106,107,108,109,110,111]. After decomposition of signals by using VMD, signal characteristics of fault are obtained by some other methods. Aneesh [90] employed support vector machine to obtain detection of faults with statistical parameter vector of IMF candidates. In addition, classification results using support vector machine shows that VMD outperforms EWT for feature extraction processes and the classification accuracy is recorded. Lv [92] adopted multikernel support vector machine optimized by Immune Genetic Algorithm to diagnose outer ring damage, rolling damage, and inner ring damage of a bearing. The experiments of mechanical faults showed that, compared to traditional fault diagnosis models, the proposed method significantly increased the diagnosis accuracy of mechanical faults and enhances the generalization of its application. Muralidharan [93] used the J48 decision tree algorithm to identify the useful features, and the selected features were used for classification using the decision trees, namely Random Forest, REP Tree and Logistic Model Tree algorithms, and the performance analyses of these algorithms were done in detail. Liu [94] presented an algorithm to extract fault features of a rolling bearing, combining singular value decomposition and standard fuzzy C means clustering. The result showed that, in comparison to a similar process based on EMD, VMD was not sensitive to the initialization of standard fuzzy C means clustering and exhibited a better classification performance in the same load fault diagnosis. Tang [95] proposed a method to solve the underdetermined problem and to extract fault features based on VMD. After decomposition of signals by using VMD, the demodulated signals with HT of these multi-channel functions were used as the input matrix for ICA to separate compound faults of roller bearings. An [98] took permutation entropy of components carrying key fault information obtained by VMD of signals as a bearing fault characteristic value, and the nearest neighbor algorithm was employed as a classifier to identify faults in a roller bearing. An [100] also used the *K* nearest neighbor algorithm to extract energy characteristic parameters from components carrying defect information decomposed by VMD to obtain fault diagnosis of rolling bearings of a wind turbine. Yang [101] employed local linear embedding to reduce the dimensionality of these extracted features extracted from both VMD sub-signals and the original one and made the samples more separable. Then, multiclass support vector machine was used to diagnose mechanical faults of a rotor-bearing-casing system. Huang [102] divided the IMF matrix obtained by using VMD into submatrices to compute the local singular values. In addition, a multi-layer classifier composed of two one-class support vector machines and a support vector machine was constructed to identify the fault type of high-voltage circuit breakers with the maximum singular values of each submatrix.

To obtain more promising decomposition results and enhance the adaptivity of the method, scholars have made improvements to VMD. Yi [103] optimized local extremum of individual particles and global extremum of group particles by using a particle swarm optimization algorithm to improve VMD. The results indicated that the method was much more robust to sampling and noise. Liu [104] improved VMD by using the correlation coefficient criterion to determine the number of mono-components adaptively. Zhu [105] employed kurtosis as an optimization index to determine the number of decomposition modes and data-fidelity constraint of VMD by using an artificial fish swarm algorithm.

As VMD can decompose a multi-component signal into different mono-components, the algorithm can separate noise from signals. Some applications and researches were done to the de-noising issue by using VMD. Zhang [96] employed majoriation–minization-based total variation denoising to eliminate stochastic noise in the raw signal. An [106] took the approximate entropy of modes obtained by using VMD as evaluation parameter of the significance of the mode for the original signal, and the de-noising signal was constructed with modes with approximate entropies greater than threshold. The results showed that the method had better de-noising performance than WTs in terms of SNR, root mean square error and partial correlation index. Liu [107] presented a criterion based on detrended fluctuation analysis to select the mode number of VMD, aiming to avoid the impact of overbinning or underbinning on the VMD denoising. Yao [108] presented a noise source identification algorithm for diesel engines based on variational mode decomposition and robust independent component analysis. After the VMD of signals, the RobustICA algorithm was employed on the modes to extract the independent components. Furthermore, the continuous wavelet transform and the prior knowledge of diesel engines were applied to further identify the separated results. Table 2 is designed to make this section more readable.

## 5. Vold–Kalman Filter Order Tracking

VKF_OT can determine the slowly-varying envelope of tracked order components with known instantaneous frequencies [112,113,114]. The algorithm was first employed in vibration analysis of rotating machinery. In this paper, to explore the theoretical details of VKF_OT technique realization and parameter characteristics, we explicitly state the mathematical background of both the angular-velocity and angular-displacement VKF_OT techniques, according to Ref. [115]. The purpose of the method is to obtain the tracked order components by minimizing the energy of errors for both the structural and data equations by mean of one of the least squares approaches [116].

### 5.1. Principle of Vold–Kalman Filter Order Tracking

#### 5.1.1. The Angular-Velocity Vold–Kalman Filter Order Tracking

##### *The* *Structural Equation*

For a second-order ordinary differential equation (ODE)

(41) $$\frac{d^{2}f(t)}{dt^{2}}+\omega^{2}f(t)=0$$

The complementary solution is

(42) $$f(t)=K_{1}e^{j\omega t}+K_{2}e^{-j\omega t}$$

where *K*_1_ and *K*_2_ are arbitrary constants. The discrete form can be expressed as

(43) $$f(t)=K_{1}e^{nj\omega \Delta t}+K_{2}e^{-nj\omega \Delta t}$$

where *t* = *n*Δ*T*; *n* = 1, 2, 3, ...; and Δ*T* denotes the sampling time spacing. Let $d_{1}=e^{j\omega \Delta T}$ and $d_{2}=e^{-j\omega \Delta T}$; respectively, then Equation (43) becomes

(44) $$f(n)=K_{1}{\left(d_{1}\right)}^{n}+K_{2}{\left(d_{2}\right)}^{n}$$

and the characteristic equation can be expressed as

(45) $$H(n)=(D-d_{1})(D-d_{2})$$

where the operator notation *D* denotes a discrete-time delay such as Df(n)=f(n−1). The analyzed signal f(n) satisfies the following second-order difference equation,

(46) f(n)−2cos(ωΔT)f(n−1)+f(n−2)=0

where f(n) denotes the tracked order component, and *ω* is the radian frequency. Generally, a non-homogeneous term, *ε*(*n*) is introduced to represent the other not-concerned components. So the amplitude, frequency and phase change slightly. In addition, Equation (46) can be written as [115]

(47) f(n)−2cos(ωΔT)f(n−1)+f(n−2)=ε(n)

Equation (47) is called the structural equation of the angular-velocity VKF_OT.

##### *The* *Data Equation*

An analyzed signal *y*(*n*) possesses a formality like [115]

(48) y(n)=f(n)+η(n)

where the component η(n) denotes other not-concerned components. Equation (48) represents the data equation of the angular-velocity VKF_OT.

##### *Computation* *of the Tracked Order Component f*

Equation (47) expresses the tracked order component, and Equation (48) expresses the measured signal. It is assumed that the length of the measured signal *y*(*n*) is *N*, and the tracked order component *f*(*n*) is calculated with Equations (47) and (48), i.e.,

(49) $$\left[\begin{array}{ccccccccc} 1 & -c & 1 & 0 & 0 & \cdots & 0 & 0 & 0\\ 0 & 1 & -c & 1 & 0 & \cdots & 0 & 0 & 0\\ 0 & 0 & 1 & -c & 1 & \cdots & 0 & 0 & 0\\ \vdots & \vdots & \vdots & \vdots & \vdots & \vdots & \vdots & \vdots & \vdots \\ 0 & 0 & 0 & 0 & 0 & \cdots & 1 & -c & 1 \end{array}\right]\left[\begin{array}{l} f(1)\;\\ f(2)\;\\ f(3)\;\\ \vdots \\ f(N)\; \end{array}\right]=\left[\begin{array}{l} \varepsilon (1)\;\\ \varepsilon (2)\;\\ \varepsilon (3)\;\\ \vdots \\ \varepsilon (N)\; \end{array}\right]$$

and

(50) $$\left[\begin{array}{l} y(1)\;\\ y(2)\;\\ y(3)\;\\ \vdots \\ y(N)\; \end{array}\right]=\left[\begin{array}{l} f(1)\;\\ f(2)\;\\ f(3)\;\\ \vdots \\ f(N)\; \end{array}\right]+\left[\begin{array}{l} \eta (1)\;\\ \eta (2)\;\\ \eta (3)\;\\ \vdots \\ \eta (N)\; \end{array}\right]$$

where c=2con(ωΔT). Equations (49) and (50) can be symbolized, respectively, as

(51) $$\overset{↔}{A}\tilde{f}=\tilde{\varepsilon }$$

and

(52) $$\tilde{y}=\tilde{f}+\tilde{\eta }$$

where the matrix $\overset{↔}{A}$ is a sparse matrix, and the dimension is (N−2)×N; $\tilde{f}$ denotes the tracked order component, and $\tilde{y}$ represents the measured data; $\tilde{\varepsilon }$ is the vector of the non-homogeneous term, and $\tilde{\eta }$ is the vector of the not-concerned component. The norm square of the non-homogeneous vector is as follows

(53) $${\tilde{\varepsilon }}^{T}\tilde{\varepsilon }={\tilde{f}}^{T}{\overset{↔}{A}}^{T}\overset{↔}{A}\tilde{f}$$

where the symbols *T* denote the transpose operations. Likewise, the norm square of the not-concerned vector can be expressed as

(54) $${\tilde{\eta }}^{T}\tilde{\eta }=({\tilde{y}}^{T}-{\tilde{f}}^{T})(\tilde{y}-\tilde{f})$$

The least squares approach is employed to calculate the tracked order component. The calculation goal is to minimize the energy of errors for both the structural and data equations. A weighting factor *r* is used to tune the tracked order component $\tilde{f}$ with desirable resolutions. A weighted combination forms by combining both the structural and data equations,

(55) $$J=r^{2}{\tilde{\varepsilon }}^{T}\tilde{\varepsilon }+{\tilde{\eta }}^{T}\tilde{\eta }$$

is employed to evaluate $\tilde{f}$; where $r^{2}{\tilde{\varepsilon }}^{T}\tilde{\varepsilon }={\tilde{f}}^{T}{\overset{↔}{A}}^{T}\overset{↔}{A}\tilde{f}$: To make $\partial J/\partial \tilde{f}=0$, the calculation result is as follows,

(56) $$(r^{2}{\overset{↔}{A}}^{T}\overset{↔}{A}+\overset{↔}{I})\tilde{f}=\tilde{y}$$

The tracked order component $\tilde{y}$ is calculated by using the LU decomposition method [117]. Every mono-component $\tilde{f}$ depends on the corresponding instantaneous amplitude and instantaneous phase, and they are considered as local constants. In addition, enough time points are needed to compute the amplitude and phase.

##### *Supplement* *to Amplitude and Phase of Tracked Order*

In Equation (47), it is assumed that the radian-frequency (*ω*) is a constant. The tracked order component $\tilde{f}$ can be calculated with known IF. In a second-order, ODE is called the angular-velocity VKF_OT, and the tracking procedure is obtained in another way. The computed order component $\tilde{f}$ is calculated by

(57) $$f(n)=\left[\cos(\sum_{m=0}^{n}\omega (m)\Delta T)\;\sin(\sum_{m=0}^{n}\omega (m)\Delta T)\right]\left[\begin{array}{l} a(n)\\ b(n) \end{array}\right]$$

where the amplitude is $\sqrt{a{\left(n\right)}^{2}+b{\left(n\right)}^{2}}$, and the phase $\tan^{-1}\left(\frac{a(n)}{b(n)}\right)$. In the next subsection, another OT technique will be explained by using a different structural equation arising directly from the order waveform similar to Equation (57).

#### 5.1.2. The Angular-Displacement Vold–Kalman Filter Order Tracking

The *k*th-order component arising from the operation of a rotary machine can be expressed as

(58) $$f_{k}(t)=a_{k}(t)\theta_{k}(t)+a_{-k}(t)\theta_{-k}(t)$$

where $a_{k}(t)$ represents the complex envelope, and $a_{-k}(t)$ is the complex conjugate of $a_{k}(t)$ to make $f_{k}(t)$ a real waveform. It is noted that $\theta_{k}(t)$ is a carrier wave, and defined as

(59) $$\theta_{k}(t)=\exp(ki\int_{0}^{t}\omega (u)du)$$

where du is the speed of the reference axle, and $\int_{0}^{t}\omega (u)du$ is the elapsed angular displacement. The discrete form of Equation (59) can then be expressed as

(60) $$\theta_{k}(n)=\exp(ki\sum_{m=0}^{n}\omega (m)\Delta T)$$

##### The Structural Equation

To obtain the tracked order component $f_{k}(t)$, the corresponding envelope $a_{k}(t)$ needs to be computed. It is assumed that $a_{k}(t)$ can be a relatively smooth polynomial with a low degree, and fulfills [114]

(61) $$\frac{d^{S}a_{k}(t)}{dt^{s}}=\psi_{k}(t)$$

where $\psi_{k}(t)$ represents a higher-degree term in $a_{k}(t)$. Likewise, the corresponding discrete forms is as follows

(62) $$\nabla^{S}a_{k}(n)=\psi_{k}(n)$$

where ∇ denotes the difference operator; the index *s* denotes the differentiation order; $\psi_{k}(n)$ denotes a combination of other spectral components and additional measurement noise.

##### *The* *Data Equation*

A measured signal *y*(*n*) is taken as a combination of several order/spectral components, $f_{k}(t)$, and measurement noise [114]

(63) $$y(n)=\sum_{k\in j}a_{k}(n)\theta_{k}(n)+\xi (n)$$

where the integral number j(=±1,±2,±3,…,and/or±K) denotes the order of spectral components to be tracked, ξ(n) represents unwanted spectral components and measurement errors. It is noted that each order/spectral component $a_{k}(n)$ of interest modulates with its corresponding carrier wave $\theta_{k}(n)$.

##### *Computation* *of the Tracked Order Component f*

In Equation (62), let *s* = 2, and data length be *N*, then the matrix form can be expressed as

(64) $$\left[\begin{array}{cccccccc} -2 & 1 & 0 & 0 & 0 & \cdots & 0 & 0\\ 1 & -2 & 1 & 0 & 0 & \cdots & 0 & 0\\ 0 & 1 & -2 & 1 & 0 & \cdots & 0 & 0\\ \vdots & \vdots & \vdots & \vdots & \vdots & \vdots & \vdots & \vdots \\ 0 & 0 & 0 & 0 & 0 & \cdots & -2 & 1 \end{array}\right]\left[\begin{array}{l} a_{k}(1)\;\\ a_{k}(2)\;\\ a_{k}(3)\;\\ \vdots \\ a_{k}(N)\; \end{array}\right]=\left[\begin{array}{l} \psi_{k}(1)\;\\ \psi_{k}(2)\;\\ \psi_{k}(3)\;\\ \vdots \\ \psi_{k}(N)\; \end{array}\right]$$

To simultaneously track multiple orders and spectral components, e.g., resonance, Equation (63) can also be extended to all tracked order components. Let

$$\begin{array}{c} \overset{↔}{M}=\left[\begin{array}{cccccccc} -2 & 1 & 0 & 0 & 0 & \cdots & 0 & 0\\ 1 & -2 & 1 & 0 & 0 & \cdots & 0 & 0\\ 0 & 1 & -2 & 1 & 0 & \cdots & 0 & 0\\ \vdots & \vdots & \vdots & \vdots & \vdots & \vdots & \vdots & \vdots \\ 0 & 0 & 0 & 0 & 0 & \cdots & -2 & 1 \end{array}\right],\overset{↔}{A}=\left[\begin{array}{l} a_{k}(1)\;\\ a_{k}(2)\;\\ a_{k}(3)\;\\ \vdots \\ a_{k}(N)\; \end{array}\right]\;\mathrm{and}\;\overset{↔}{Z}=\left[\begin{array}{l} {\tilde{\psi }}_{k}(1)\;\\ {\tilde{\psi }}_{k}(2)\;\\ {\tilde{\psi }}_{k}(3)\;\\ \vdots \\ {\tilde{\psi }}_{k}(N)\; \end{array}\right] \end{array}$$

and then Equation (64) becomes

(65) $$\left[\begin{array}{ccccccc} \overset{↔}{M} & 0 & 0 & 0 & \cdots & 0 & 0\\ 0 & \overset{↔}{M} & 0 & 0 & \cdots & 0 & 0\\ 0 & 0 & \overset{↔}{M} & 0 & \cdots & 0 & 0\\ \vdots & \vdots & \vdots & \vdots & \vdots & \vdots & \vdots \\ 0 & 0 & 0 & 0 & \cdots & 0 & \overset{↔}{M} \end{array}\right]\overset{↔}{A}=\overset{↔}{Z}$$

where elements ${\tilde{a}}_{k}$ in the matrix $\overset{↔}{A}$ are column vectors with a length *N*, which denote the *k*th order component; ${\tilde{\psi }}_{k}$ represent error vectors with a dimension *N* × 1; and *M* is a matrix with a dimension *N* × *N*.

The terms with negative indexes in Equation (63) assure $f_{k}(t)$ to be a real waveform. $\tilde{y}$ denote the measured signal with a length of *N*, ξ an error vector with dimension *N* × 1; and ${\overset{↔}{B}}_{k}$ consist of carrier signals, which is a diagonal matrix, as

(66) $${\overset{↔}{B}}_{k}=\left[\begin{array}{ccccc} \theta_{k}(1) & 0 & 0 & \cdots & 0\\ 0 & \theta_{k}(2) & 0 & \cdots & 0\\ 0 & 0 & \theta_{k}(3) & \cdots & 0\\ \vdots & \vdots & \vdots & \vdots & \vdots \\ 0 & 0 & 0 & \cdots & \theta_{k}(N) \end{array}\right]\overset{↔}{A}=\overset{↔}{Z}$$

Thus, Equation (63) can be rewritten as

(67) $$\tilde{y}-\left[{\tilde{B}}_{1}\;{\tilde{B}}_{2}\;{\tilde{B}}_{3}\ldots {\tilde{B}}_{k}\right]\left[\begin{array}{l} {\tilde{a}}_{1}\;\\ {\tilde{a}}_{2}\\ {\tilde{a}}_{3}\;\\ \vdots \\ {\tilde{a}}_{K}\; \end{array}\right]=\tilde{\xi }$$

As the angular-velocity VKF_OT scheme, a weighting factor is introduced, and combine Equations (64) and (67), and then

(68) $$\left[\begin{array}{l} 0\;\\ 0\\ 0\;\\ \;\vdots \\ 0\\ \tilde{y}\; \end{array}\right]-\left[\begin{array}{llllll} r\overset{↔}{M} & 0 & 0 & \cdots & 0 & 0\\ 0 & r\overset{↔}{M} & 0 & \cdots & 0 & 0\\ 0 & 0 & r\overset{↔}{M} & \cdots & 0 & 0\\ \vdots & \vdots & \vdots & \vdots & \vdots & \vdots \\ 0 & 0 & 0 & \cdots & 0r\overset{↔}{M}\\ {\tilde{B}}_{1} & {\tilde{B}}_{2} & {\tilde{B}}_{3} & \ldots & {\tilde{B}}_{k-1} & {\tilde{B}}_{k} \end{array}\right]\left[\begin{array}{l} {\tilde{a}}_{1}\;\\ {\tilde{a}}_{2}\\ {\tilde{a}}_{3}\\ \;\vdots \\ {\tilde{a}}_{K-1}\\ {\tilde{a}}_{K}\; \end{array}\right]=\left[\begin{array}{l} r\overset{↔}{Z}\\ \xi \end{array}\right]$$

For the convenience of subsequent deviation, Equation (68) can be symbolized as

(69) $$\overset{↔}{Y}-\overset{↔}{P}\overset{↔}{A}=\overset{↔}{E}$$

The evaluation of tracked order components is exactly to find a vector $\overset{↔}{A}$ fulfilling

(70) $$\underset{\overset{↔}{A}}{\min}({\left\|\overset{↔}{E}\right\|}^{2})=\underset{\overset{↔}{A}}{\min}({\overset{↔}{E}}^{H}\overset{↔}{E})=\underset{\overset{↔}{A}}{\min}(J)$$

i.e., $\partial J/\partial \overset{↔}{A}=0$. The vector $\overset{↔}{A}$ can be written as

(71) $${\overset{↔}{P}}^{H}\overset{↔}{P}\overset{↔}{A}={\overset{↔}{P}}^{H}\overset{↔}{Y}$$

The matrix ${\overset{↔}{P}}^{H}\overset{↔}{P}$ is of the form

(72) $${\overset{↔}{P}}^{H}\overset{↔}{P}=\left[\begin{array}{ccccc} \overset{↔}{S} & {\overset{↔}{B}}_{1,2} & {\overset{↔}{B}}_{1,3} & \cdots & {\overset{↔}{B}}_{1,K}\\ {\overset{↔}{B}}_{2,1} & \overset{↔}{S} & {\overset{↔}{B}}_{2,3} & \cdots & {\overset{↔}{B}}_{2,K}\\ {\overset{↔}{B}}_{3,1} & {\overset{↔}{B}}_{1,2} & \overset{↔}{S} & \cdots & {\overset{↔}{B}}_{3,K}\\ \vdots & \vdots & \vdots & \ddots & \vdots \\ {\overset{↔}{B}}_{K,1} & {\overset{↔}{B}}_{K,2} & {\overset{↔}{B}}_{K,3} & \cdots & \overset{↔}{S} \end{array}\right]$$

where $\overset{↔}{S}=r^{2}{\overset{↔}{M}}^{T}\overset{↔}{M}+\overset{↔}{I}$, and ${\overset{↔}{B}}_{u,v}=B_{u}^{H}B_{v}$, Moreover, ${\overset{↔}{P}}^{H}$ is of the form

(73) $${\overset{↔}{P}}^{H}={\left[{\bar{\overset{↔}{B}}}_{1}\;{\bar{\overset{↔}{B}}}_{2}\;{\bar{\overset{↔}{B}}}_{3}\cdots \;{\bar{\overset{↔}{B}}}_{K}\right]}^{T}$$

where ${\bar{\overset{↔}{B}}}_{K}$ denotes the complex conjugate of ${\overset{↔}{B}}_{K}$. It should be noted that Equation (72) is positive definite, its inverse matrix exists and can be evaluated numerically.

### 5.2. Advantage of Vold–Kalman Filter Order Tracking

**Figure 39:**
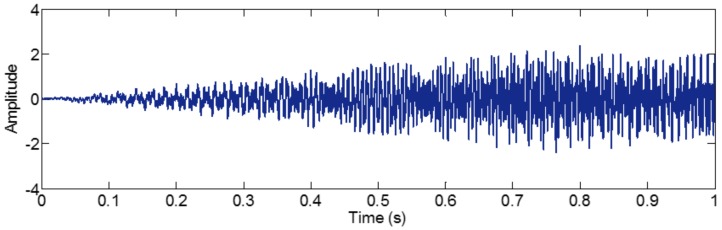
The waveform of the sample signal *f_sig_*_8_.

**Figure 40:**
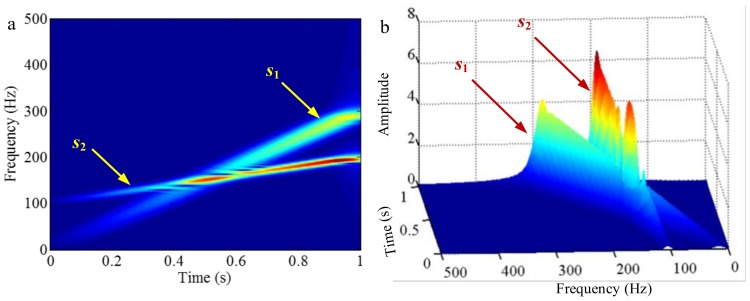
The STFT of the sample signal *f_sig_*_8_: (**a**) 2D figure and (**b**) 3D figure.

**Figure 41:**
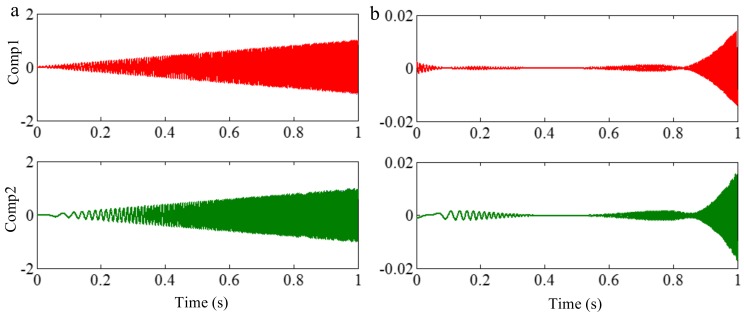
The waveforms of decomposition result of *f_sig_*_8_ by using VKF_OT and the corresponding calculation errors: (**a**) waveforms and (**b**) calculation errors.

As mentioned above, the different mono-components can be separated by using VKF_OT with known IF, even when the IFs cross in time-frequency panel, which cannot be done by methods deriving from EMD, EWT and VMD. We construct a sample *f_sig_*_8_ (as shown in Figure 39)

(74) $$f_{sig8}=s_{1}(t)+s_{2}(t)\;s_{1}(t)=t\cdot \sin[(100+100t)\times 2\pi t],0\leq t\leq 1\;s_{2}(t)=t\cdot \sin[(300t)\times 2\pi t],0\leq t\leq 1$$

to illustrate it, and the sampling frequency is 2 kHz. As can be seen in Figure 40, two components cross in time-frequency panel. Figure 41a presents the decomposition result. To check the calculation accuracy of the VKF_OT, we calculate the error $e_{k}(t)$ by

(75) $$e_{k}(t)=f_{k}(t)-f_{k0}(t)$$

where $f_{k}(t)$ denote the *k*th component of decomposition result (k=1,2,…, *k* is the number of components obtained by using VKF_OT), and $f_{0k}(t)$ denote the *k*th component of original signal. As shown in Figure 41b, the errors are small. Therefore, for the slowly-varying envelope of tracked order components with known instantaneous frequencies, a promising calculation result can be obtained by using VKF_OT.

### 5.3. Disadvantage of Vold–Kalman Filter Order Tracking

**Figure 42:**
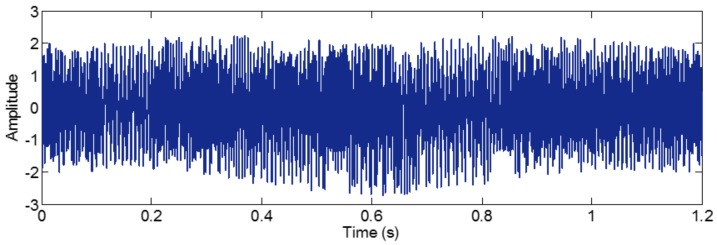
The waveform of the sample signal *f_sig_*_9_.

**Figure 43:**
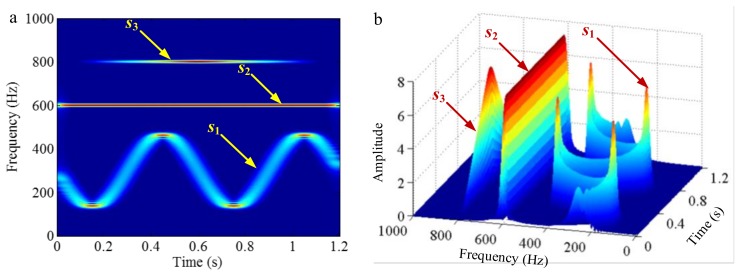
The STFT of the sample signal *f_sig_*_9_: (**a**) 2D figure and (**b**) 3D figure.

**Figure 44:**
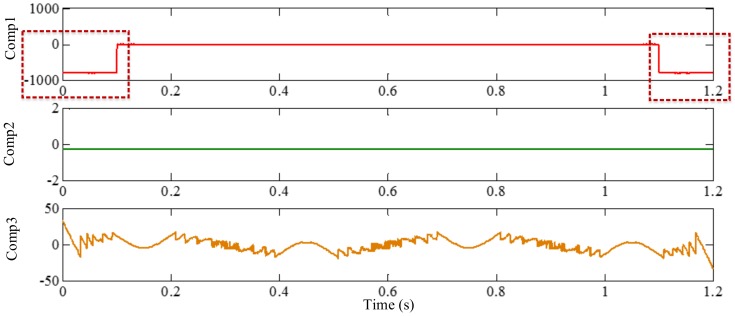
The IF errors of the sample signal *f_sig_*_9_ obtained from the corresponding STFTs.

**Figure 45:**
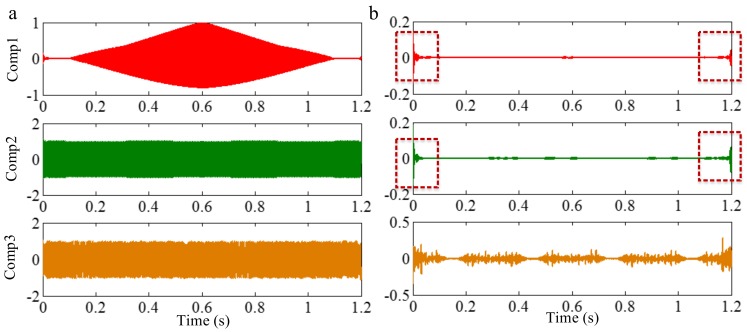
The waveforms of decomposition result of *f_sig_*_9_ by using VKF_OT and the corresponding calculation errors: (**a**) waveforms and (**b**) calculation errors.

We construct a sample signal *f_sig_*_9_ (as shown in Figure 42)

(76) $$\begin{array}{c} f_{sig8}=s_{1}(t)+s_{2}(t)+s_{3}(t)\\ s_{1}=\left\{ \begin{array}{ll} 0 & ,\;0\leq t<0.1\\ 2(t-0.1)\sin\{800\cdot 2\pi \cdot t\} & ,\;0.1\leq t<0.6\\ -2(t-1.1)\sin\{800\cdot 2\pi \cdot t\} & ,\;0.6\leq t<1.1\\ 0 & ,\;1.1\leq t<1.2 \end{array}\right.\\ s_{2}(t)=t\cdot \sin[(600t)\times 2\pi t],0\leq t\leq 1.2\\ s_{3}=\sin[300\cdot 2\pi \cdot t+100\sin(2\pi t/1.2)],0\leq t\leq 1.2 \end{array}$$

to demonstrate the disadvantage of VKF_OT, and the sampling frequency is 2 kHz. In this paper, we employ STFT to obtain the IF of each component. On this basis, VKF_OT is adapted to compute components of the signal. The STFT of the sample signal *f_sig_*_9_ is shown in Figure 43. The IF errors of the sample signal *f_sig_*_9_ is obtained from the corresponding STFTs, and is presented in Figure 44. In addition, the calculation result of VKF_OT is presented in Figure 45. As shown in Figure 45, the computation error of components 1 and 2 are small, except for the signal ends marked by red rectangles in Figure 45. However, the errors of component 3 are relatively greater, about 0.1, which may result from the error of IF. As shown in Figure 44, the IF errors of component 3 are relatively greater, too. The IF of component 3 varies relatively quickly, so it is difficult to obtain IF with a high accuracy by using STFT. It can be deduced that a high computation accuracy of the component 3 can be obtained, if the IF difference between the calculation result and the true value is small. Moreover, as can be seen in Figure 44, the IF errors of component 1 in [0 0.1] s and [1.1 1.2] s are great, as marked by red rectangles in Figure 44, but the calculation errors in these time sections are also small. Therefore, this phenomenon reveals that the error of IF cannot result in calculation error when the values of the component are zero in the corresponding time sections.

The analysis above indicates that precisely calculating IF is crucial for the calculation accuracy of VKF_OT. In this paper, STFT is adapted. Maybe, other time-frequency representation techniques can be tried to obtain the IF of component with a high accuracy. The selection of parameters such as the weighting factor and the correlation matrix of process noise, which influences tracking performance, are issues open to further research.

### 5.4. Application and Improvement Works of Vold–Kalman Filter Order Tracking

As illustrated above, VKF_OT can single out mono-component-related signatures, so it is an effective tool for the analysis of measured dynamic signals. Scholars have done numerous investigations of application and improvement research for VKF_OT [113,114,115,116,117,118,119,120,121,122,123,124,125,126,127,128,129,130,131,132,133,134,135].

First, we summarize some theoretical researches of VKF_OT. Vold [114] proposed VKF_OT for the estimation of a single-order component. Afterwards, an improved version simultaneously estimating multiple orders was proposed [113,114]. Pan [115,116] further explored the theoretical details of the angular-velocity and angular-displacement VKF_OTs. However, these VKF_OT schemes must be computed off-line and implemented as post-processing techniques, resulting from determination of structural equations and data equations [113,114], which makes the unknown complex envelopes smooth and relates the tracked orders to the measured signal. These two equations should be evaluated within a huge inverse matrix with all observed time sequence data. The solution of Kalman filtering converges to the optimum Wiener solution in some statistical sense. It can be seen that embodying the structural and data equations of a linear, discrete-time dynamical system in the process and measurement equations translates the order-tracking problem into a state estimation task. Haykin [118] introduced a one-step prediction into Kalman filtering, overcoming this drawback of the original VKF_OT scheme, and made real-time processing feasible. In addition, Wu [119] employed the algorithm to undertake fault diagnosis of a gear set and damaged engine turbocharger wheel blades. In Ref. [119], sound emission signals served as an alternative reference signal to the fault diagnosis system. Pan [120,121] took this and improved the original angular-velocity and angular-displacement VKF_OTs [115,116], which enabled addressing of computation complexity, and allowed it to be considered in on-line and real-time applications. Pan [122] adopted the procedure of accumulative vectors and the concept that a measured signal could be represented as the superposition of order components to the original angular-velocity Vold–Kalman order tracking [115,116], and presented an extended angular-velocity VKF_OT. It is worth mentioning that Pan [128] built a remote online machine condition monitoring system in the architecture of both the Borland C++ Builder (BCB) software-developing environment and Internet transmission communication. Various signal-processing computation schemes such as time–frequency analysis and VKF_OT were implemented-based upon the Borland C++ Builder graphical user interface.

To improve performance of the method for the dynamics analysis, other signal processing algorithms were employed to analyze testing signals together with VKF_OT. Wang [123] used EMD to preprocess raw signals, and then further decompose IMFs to separate speed synchronous and non-synchronous vibrations by using VKF_OT. Besides, to select a suitable bandwidth of VKF_OT in implementation of Vibration Monitoring of Electrical Machines, Wang [124] established a simplified simulation model of electrical rotating machinery, and a parameter was chosen based on two different damping ratios of the simulation model. Similar to Ref. [123], Guo [125] applied ICA to decouple the disturbance orders. Furthermore, the independent components were decomposed using VKF_OT. Feng [126] employed higher-order energy separation on mono-components obtained by VKF_OT to accurately estimate the IF because of its high adaptability to local signal changes.

## 6. Summary and Prospects

Adaptive methods to analyze a signal are of great interest regarding finding sparse representation in the contest of compressive sensing. Employing a proper adaptive decomposition algorithm tends to successfully separate a multicomponent signal into different mono-components. Practical engineering problems can be roughly divided into two categories, precise filtering operations and rough filtering operations. The former requires that a single targeted mono-component should be accurately extracted form a raw signal, and it is ideal that there should be no loss of the targeted mono-component and no residual noise. The parameter identification of mechanical systems [136,137] and isolation of deferent wave packages in ultrasonic non-destructive testing [67] belong to this category. For the latter, highlighting specific characteristics of valuable components is the filter target, and the loss of the valuable component and the residual noise (invaluable components can be taken as noise in this paper) are tolerable, for example in fault diagnosis of rolling bearings [138,139,140]. The algorithms mentioned above can be taken as different filters, and have their respective applicable scopes, inapplicable scopes and further research issues, as summarized in Table 3 and Table 4. In real applications, one should select an appropriate method according to the specific characteristics of the signal.

### 6.1. Algorithms Deriving from Empirical Mode Decomposition

EMD decomposes multicomponent signals in sifting and iteration process. EEMD solves mode-mixing caused by intermittence signals. CEEMD can suppress the residue coming from adding white noise in the decomposition process. CEEMDAN and improved CEEMDAN can reduce computation amount.

EEMD, CEEMD, CEEMDAN and improved CEEMDAN can work when the IFs of different mono-components are distinct enough at each time point. It is necessary for the ratio between a relatively low IF and a relatively high IF to be smaller than 0.75, and an ideal decomposition result can be obtained when the ratio is smaller than 0.5, for reasonable numbers of sifting iterations. For the precise filtering operation mentioned above, this necessary condition of the frequency resolution should be met; otherwise, a relatively large calculation error may be introduced. Further work can be done on decreasing computation intensity and improving decomposition stability.

For the rough filtering operation mentioned above, algorithms deriving from EMD can work in most cases. If the conduction of frequency resolution is not met, although the loss of valuable components and residual noise may occur, noise can be removed to a certain degree by using algorithms deriving from EMD. Therefore they may work in this case. Moreover, algorithms deriving from EMD can be employed in conjunction with other decomposition methods such as wavelet transforms, principal component analysis and adaptive multiscale morphological analysis to further remove color noise and highlight the specific characteristics of the valuable component. In addition, identifying interesting components is also a research issue. Finally, further work can be done on decreasing computation intensity and improving decomposition stability.

### 6.2. Empirical Wavelet Transform

EWT is a combination of WT and EMD. For precise filtering operations, the necessary premise is that valuable mono-components can be separated in the Fourier spectrum. However, considering the characteristics of the Fourier spectrum in different practical problems as shown in Section 3.3.2, the filtering goal can be met under the condition that the boundaries of valuable mono-components can be obtained; therefore suitable strategies for boundary detection is crucial. Further research can be conducted with respect to this problem. White noise distributes in the entire Fourier spectrum. For a broadband mono-component, the negative effect from white noise cannot be neglected in some situations. In that case, a de-noising operation before or after EWT may be necessary. Further work may be done by finding effective de-noising methods for this problem.

For the rough filter operation, EWT can work in most cases. Similar to the precise filter operation, correctly establishing the boundary of valuable components is decisive. Therefore, finding out the spectrum band corresponding to the valuable component in different practical problems is an open research issue. A specific effective boundary detection strategy can be taken as a significant contribution for a scientific problem. Finally, further removing color noise in conjunction with other decomposition methods, highlighting the specific characteristics of the valuable component, and identifying interesting components also are research issues for EWT.

### 6.3. Variational Mode Decomposition

VMD decomposes a multicomponent signal into a series of sub-signals (mono-component) that have specific sparsity properties by assessing the bandwidth of a mono-component in an iteration process using an ADMM. For the precise filter operation, as shown in Section 4.2, it is necessary that the different mono-components are well isolated in the frequency spectrum of the raw signal. Otherwise, decomposition cannot be successful. Further, the sparsity property employed in VMD is that the mono-component should be mostly compact around a center pulsation in the frequency spectrum. The widespread application of original VMD seems to suggest that this goal has fine applicability. However, in practical problems, this goal may not be universal in all cases. A suitable sparsity property should be defined for a specific problem. In addition, the frequency spectrum can be extended into time-frequency spectrums. Similar to EWT, the effluence of white noise should also be taken into consideration, as it is inevitable that each bandwidth of the frequency spectrum keeps part of the energy of white noise. Therefore, a de-noising operation before or after VMD may be necessary, if the white noise has a strength of energy that cannot be ignored. In addition, the selection of parameters such as number of decomposition modes and data-fidelity constraint, which influences tracking performance, is an issue open to future research.

For rough filter operation, VMD can work in most cases. The goal of extracting the interesting component from the raw signal can also be obtained, when different mono-components overlap in the corresponding frequency spectrum useless components are inevitable. Similar to the precise filter operation, finding a suitable sparsity property in a specific practical problem and the selection of parameters are open issues. In addition, the selection of parameters is also an issue open to future research. Finally, further removing color noise in conjunction with other decomposition methods, highlighting the specific characteristics of the valuable components and identifying interesting components also are research issues for VMD.

### 6.4. Vold–Kalman Filter Order Tracking

VKF_OT can decompose multicomponent signals into different mono-components with known corresponding IFs. Therefore, VKF_OT is more suitable for precise filter operation, compared with rough filter operation. Because the calculation accuracy depends on the accuracy of the IF, calculating the IF at a high accuracy is key for VKF_OT. We can employ time-frequency analysis techniques. However, when the IF changes quickly, it is difficult to obtain the IF precisely. Recently, some novel time-frequency representation techniques such as polynomial chirplet transform [141,142] and synchrosqueezing transform [143] have become available. These methods may be ideal choices to obtain the IF. Obtaining the IF to a high accuracy and the selection of parameters such as the weighting factor and the correlation matrix of process noise are issues open to future research.

## Figures and Tables

**Table 1:** Summary of works for EMD.

Objects		References	Methodologies
Precise filter operation	Non-linear system	Lee et al. [30]	EMD
		Chen et al. [33]	
		Poon et al. [34]	
	Beam with a bolted joint connection	Eriten et al. [31]	
	Non-linear system	Yang et al. [26,27]	EMD + HT
		Pai et al. [29]	
	Aerial Planting Projectile flight data	Goodarzi et al. [32]	
	Non-linear system	Pai et al. [35]	EMD + conjugate-pair decomposition method
	Cable-stayed bridge	Khan et al. [28]	EEMD + Pareto technique
Rough filter operation	Bear	Van et al. [37]	EMD + non-local-means de-noising + particle swarm optimization + *K*-nearest neighbors, probabilistic neural network and support-vector machine
		Ali et al. [39]	EMD + artificial neural network
		Georgoulas et al. [41]	EMD + HT
		Georgoulas et al. [42]	EMD + HT+ hidden Markov model
		Meng et al. [50]	EMD + hidden Markov model classifier
		Zhao et al. [45]	EMD + the approximate entropy method
		Djebala et al. [46]	EMD + optimized wavelet multi-resolution
		Saidi et al. [47]	EMD + Bi-spectrums, a third-order statistic
		Wang et al. [49]	EMD + ICA
		Zhang et al. [40]	EEMD + support-vector machine
		Zheng et al. [51]	GEMD + EED
		Zheng et al. [53]	PEEMD
	High-speed train	Bustos et al. [36]	EMD
	Centrifugal pumps	Wang et al. [38]	CEEMD + random forest classifier
	Gear	Le et al. [48]	EMD + radial basis function neural network
	Rotating machinery	Jiang et al. [54]	EEMD + multiwavelet packet

**Table 2:** Summary of works for VMD.

Objects			References	Methodologies
Precise filter operation	speech signal		Upadhyay et al. [84]	VMD
			Upadhyay et al. [85]	
	X-band open-ended rectangular waveguide		Yin et al. [86]	
	metal burn degrees		Gao et al. [87]	
	ship-radiated noise		Li et al. [88]	VMD + support vector machine
Rough filter operation	Bearings		Lv et al. [92]	VMD + multikernel support vector machine
			Liu et al. [94]	VMD + singular value decomposition and standard fuzzy C means clustering
			Tang et al. [95]	VMD + HT + ICA
			An et al. [98]	VMD + nearest neighbor algorithm
			An et al. [100]	VMD + *K* nearest neighbor algorithm
			Yang et al. [101]	VMD + multiclass support vector machine
	Power signal		Aneesh et al. [90]	VMD + support vector machine
	Gear		Muralidharan et al. [93]	VMD + J48 decision tree algorithm
	Voltage circuit breaker		Huang et al. [102]	VMD + one-class support vector machine
	Improving VMD	Bearings	Yi et al. [103]	VMD + particle swarm optimization
			Zhu et al. [105]	VMD + artificial fish swarm
		Rotor system	Liu et al. [94]	VMD + correlation coefficient criterion
	De-noising	Bearings	Zhang et al. [96]	VMD + majoriation–minization-based total variation
		Hydropower unit	An et al. [106]	VMD + approximate entropy
		Bumps, Blocks, Heavysine, Doppler and ECG	Liu et al. [107]	VMD + detrended fluctuation analysis
		Diesel engine	Yao et al. [108]	VMD + robust independent component analysis

**Table 3:** Comparison of various time–frequency analysis methods for the precise operation. It should be noticed that algorithms deriving from EMD that are referred in this paper include EEMD, CEEMD, CEEMDAN, and improved CEEMDAN, and the number of sifting iterations is set as 2000, and the stopping criterion threshold is set as 0.05. The conclusion about algorithms deriving from EMD is obtained under these conditions of parameter setting.

Method	Applicable Scope	Inapplicable Scope	Further Work
Algorithms deriving from EMD	The IFs of different component are in an enough distinction degree.	The ratio is greater than 0.75. In addition, the ratio (low IF/high IF) is less than 0.5, which can obtain a promising decomposition.	1. Decrease computation intensity; 2. Improve decomposition stability.
EWT	Different components can be separated in Fourier spectrum.	Different components overlap in Fourier spectrum.	1. Develop detection strategy of boundary; 2. Suppress influence of the white noise.
VMD	Different components can be separated in Fourier spectrum.	Different components overlap in Fourier spectrum.	1. Develop the specific sparsity property; 2. Suppress influence of the white noise; 3. Select parameters such as number of decomposition modes and data-fidelity constraint.
VKF_OT	The component IF is available.	The component IF is unknown. In addition, the calculation accuracy of mono-component depends on the precision of corresponding IF.	1. Obtain the IF in a high precision; 2. Select parameters such as the weighting factor and the correlation matrix of process noise.

**Table 4:** Comparison of various time–frequency analysis methods for the rough operation. It should be noticed that algorithms deriving from EMD that are referred in this paper include EEMD, CEEMD, CEEMDAN, and improved CEEMDAN, and the number of sifting iterations is set as 2000, and the stopping criterion threshold is set as 0.05. The conclusion about algorithms deriving from EMD is obtained under these conditions of parameter setting. Because algorithms deriving from EMD, EWT and VMD can work for most cases for the rough operation, we just list the further works for them.

Method	Further Work
Algorithms deriving from EMD	1. Decrease computation intensity; 2. Improve decomposition stability; 3. Further remove color noise in conjunction with other decomposition methods; 4. Highlight the specific characteristics of the valuable component; 5. Identify interesting components.
EWT	1. Develop detection strategy of boundary; 2. Further remove color noise in conjunction with other decomposition methods; 3. Highlight the specific characteristics of the valuable component; 4. Identify interesting components.
VMD	1. Develop the specific sparsity property; 2. Select parameters such as number of decomposition modes and data-fidelity constraint; 3. Further remove color noise in conjunction with other decomposition methods; 4. Highlight the specific characteristics of the valuable component; 5. Identify interesting components.

## Abbreviations

EMD	empirical mode decomposition
EWT	empirical wavelet transform
VMD	variational mode decomposition
VKF_OT	Vold-Kalman filter order tracking
EEMD	ensemble empirical mode decomposition
CEEMD	complementary ensemble empirical mode decomposition
CEEMDAN	complementary ensemble empirical mode decomposition with adaptive noises
Improved CEEMDAN	improved complementary ensemble empirical mode decomposition with adaptive noises
AM	amplitude modulated
FM	frequency modulated
IF	instantaneous frequency
IA	instantaneous amplitude
HT	Hilbert transform
STFT	short time Fourier transform
IMF	intrinsic mode functions
SNR	signal-noise ratio
HHT	Hilbert-Huang Transform
ICA	independent component analysis
GEMD	generalized empirical mode decomposition
EED	empirical envelope demodulation
PEEMD	partly ensemble EMD
FT	Fourier transform
ADMM	alternate direction method of multipliers
